# The potential applications of peptide-loading complex in cancer treatment

**DOI:** 10.3389/fimmu.2025.1526137

**Published:** 2025-03-03

**Authors:** Zhidu Song, Ying Tao, Jiaxin You

**Affiliations:** ^1^ Department of Ophthalmology, The Second Hospital of Jilin University, Changchun, China; ^2^ Department of Anesthesiology, China-Japan Union Hospital of Jilin University, Changchun, China

**Keywords:** peptide-loading complex, cancer, immunotherapy, major histocompatibility complex (MHC), cancer vaccine, treatment

## Abstract

Immunotherapy for cancer has made significant strides in the last several years. The prognosis for cancer patients has significantly improved as a result, particularly in hematological diseases. However, it was discovered that translating these achievements to solid tumors proved challenging. The peptide-loading complex (PLC), a temporary multisubunit membrane assembly in the endoplasmic reticulum (ER), is crucial for initiating a hierarchical immune response. Chaperones calreticulin and tapasin make up the PLC, unique to class I glycoproteins, thiooxido-reductase ERp57, and a transporter associated with antigen processing. The loading and editing of major histocompatibility complex class I (MHC-I) molecules with peptide translocation into the ER are synchronized by the PLC. One of the immune escape strategies revealed for tumors so far is changes in the expression of MHC molecules. This is because MHC antigens are crucial in presenting antigens to T-lymphocytes and controlling NK cell activity. Furthermore, decreased MHC-I expression has been linked to malignancies resistant to T-cell-based cancer immunotherapies (adoptive transfer of antitumor CD8 T-cells or checkpoint inhibition). The PLC is essential for T-cell priming, differentiation, and tumor growth control because it can bind to a wide range of MHC-I allomorphs. In this review, we have looked into PLC’s function and effects in all forms of cancer to improve cancer therapy techniques.

## 1 Introduction

Cancer affects people of all ages, socioeconomic levels, and ethnicities, making it a global health threat (1, 2). Cancer treatments include hormone therapy, radiation, chemotherapy, and surgical excision of the cancerous tissue (3). One popular method of treating cancer is chemotherapy, which involves the systemic administration of anticancer medications to patients to slow the unchecked growth of cancer cells (4). The effectiveness of immune checkpoint inhibitors in treating lung cancer (LC) (5) and melanoma (6) has sparked research into immunotherapy for a variety of tumor forms, including breast cancer (BC) (7). However, these trials have shown only a modest clinical benefit in many scenarios (8, 9). Therefore, to develop innovative treatment approaches that boost antitumor immunity in more resistant tumors, it is crucial to comprehend normal immunity and clarify resistance mechanisms that contribute to the evolution of cancer (3, 10, 11).

The capacity of immunotherapy to provide long-term effects in a substantial number of patients is crucial to its prospects. T-cell-based immunotherapeutic approaches, such as adoptive transfer of TCR-engineered T-cells and personalized cancer vaccines, can improve outcomes compared to immune checkpoint inhibition, which has demonstrated low rates of long-term tumor regression in individuals with recurring cancer (12). To implement these methods, T-cell antigen determination is necessary. Finding these antigens has been limited to researching a few tumor forms, mainly melanoma, in the most prevalent human leukocyte antigen (HLA) alleles (13). Although true immunogenic epitopes appear to be rare, recent advances in epitope discovery have the potential to open the door to more thorough protein and mutation searches, which could pave the way for tailored cancer immunotherapy for patients whose tumors retain all of their antigen and processing machinery (14). Unfortunately, there are many limitations with researchers’ current technology that make it hard to reliably identify an antigen that has been processed and presented in nature. According to recent research, epitope discovery has the potential to cause significant tumor shrinkage in individuals with advanced metastases (14). Future advancements in cancer treatment could be significantly aided by creating methods that facilitate precise and efficient antigen discovery, such as integrating peptide: HLA complex stability, which are essential components of TCR discovery for personalized cancer vaccine therapy and adoptive T-cell transfer (15–17).

Immunological investigations examine numerous immune responses, routes, control, recognition, and specificity, providing essential information for manipulating treatment outcomes (18). By using structural determination techniques, principally cryo-electron microscopy (cryoEM) or X-ray crystallography, on ligand-receptor complexes and different multiprotein assemblies, such as the peptide-loading complex (PLC), researchers may enhance their comprehension of functions and processes. A primary objective of structural immunology is to use this structural data to develop novel pharmaceuticals, therapies, immunogens, and vaccines (19).

Immunogenic peptides for cytotoxic CD8 + T-cell responses against cancerous or infected cells are provided by the major histocompatibility class I (MHC-I) molecules HLA (20). The MHC-I heavy chain consists of three extracellular domains called α chain which have three subunits α1, α2, α3, a transmembrane domain, and a cytoplasmic domain. The MHC-I antigen processing and presentation pathway selects peptides typically generated by proteasomal breakdown. In this pathway, two homologous peptide editors, TAPBPR and tapasin (Tsn), regulate and optimize the peptide repertoire before delivering it to the cell surface (21). The peptide transporter linked to antigen processing, ERp57, calreticulin (CRT), and MHC-I are all pieces of the puzzle in Tsn, a multiprotein PLC. Interactions within the PLC enable Tsn to load peptides onto MHC-I and keep MHC-I in a state that’s ready to receive peptides (22). A recent discovery is the Tsn-related molecule TAPBPR, which functions independently of the PLC. It is possible for TAPBPR to directly catalyze peptide exchange on MHC-I, in addition to recruiting UDP-glucose: glycoprotein glucosyltransferase 1 (UGT-1), which then reglucosylates peptide-receptive MHC-I and recycles it back to the PLC for Tsn-mediated peptide acquisition (23–26). In the PLC, TAPBPR and Tsn work together dynamically to contact MHC-I over a comprehensive interface with the MHC H chain and β2 microglobulin (β_2_m), influencing the global structure of MHC molecules and producing MHC molecules that are either peptide-free or peptide-receptive (27).

The origin of antigens meant for transfer onto MHC-I molecules consists of viral proteins or modified proteins, including oncogenes. Proteases produce peptides consisting of 8 to 10 amino acids within the cytoplasm, which are subsequently transported to the ER. The PLC plays a crucial role in the maintenance of MHC-I peptide assembly (28). The PLC consists of seven specific subunits that are meticulously organized within a singular macromolecular complex: the oxidoreductase ERp57, the chaperones Tsn and CRT, the nascent MHC-I molecule (a heterodimer made up of the α chain and β-microglobulin), the transporter associated with antigen processing (TAP) (comprising two subunits, TAP1 and TAP2, collectively known as TAP), and the oxidoreductase ERp57 (29). TAP plays a crucial role in the movement of antigens from the cytosol to the ER. PLC plays a vital role in stabilizing nascent MHC-I molecules, which remain unstable until the peptide antigen is precisely inserted into the antigen-binding cleft. When antigen peptides are correctly loaded, MHC-I molecules can exit the ER and arrive at the plasma membrane through the secretory pathway (30, 31).

The regulation of the immune system and the surveillance of cells that have undergone malignancy or infection depend on the display of antigenic peptides on MHC-I molecules. Peptides are moved from the cytosol to the ER, where peptide loading occurs through catalytic processes. The PLC verifies the accuracy of peptide-MHC-I (pMHC-I) complexes (32). The PLC oversees the complex process of generating pMHC-I complexes (33). As the complexes’ proofreader, the PLC is crucial for displaying kinetically stable pMHC-I complexes. Because MHC-I molecules linger in the secretory and recycling routes, pMHC-I stability is affected by suboptimal low-affinity peptide loading. Mutations, downregulation, or deletion of PLC components may change pMHC-I production, leading to a reduction in MHC-I molecules on the cell surface. Many different types of cancer cells and viruses employ this strategy to evade the immune system (29, 34). The PLC plays a critical role in the adaptive immune system by coordinating the transfer of peptides, loading them onto MHC-I, and optimizing their presentation. Two MHC-I-dedicated enzymes, Tsn, and the PLC-independent TAPBPR, choose stable pMHC-I complexes during peptide editing or proofreading and chaperone empty or poorly loaded MHC-I. This process is known as peptide loading and optimization. Recent structural and functional investigations of peptide editing have significantly advanced researchers’ understanding of this critical step in antigen processing and presentation (35).

In this review, we have summarized PLC and its elements. Subsequently, we have investigated the function of PLC and PLC components in different cancer types to improve cancer therapy techniques. We have now covered the shortcomings and prospects for this field of study in terms of clinical advancement and the creation of new cancer therapy approaches.

## 2 Peptide-loading complex

The ER is the site of peptide attachment to MHC-I. In addition to the MHC-I-β_2_m dimer, many other components are necessary for efficient peptide binding (33). These comprise Tsn, a membrane protein encoded by MHC, and the two antigen presentation transporter subunits (TAP1 and TAP2) critical for peptide entry into the ER from the cytosol. A massive multisubunit ER complex containing TAP and Tsn comprises MHC-I-β_2_m dimers before peptide binding. This complex also includes two soluble “housekeeping” proteins, CRT and ERp57, which are thiol oxidoreductases, in addition to these specialized components (36).

Most nucleated cells have MHC-I complexes on their surface that display peptides from both foreign and self-intracellular proteins. The formed heterotrimeric complexes comprise a polymorphism glycosylated HC, non-polymorphic β_2_m, and a peptide with an average length of nine amino acids (37). A multitude of chaperone molecules aid in assembling the class I complexes, which take place in the ER. The PLC, a multimolecular unit, is essential to this procedure. The PLC comprises the glycoprotein chaperone CRT, the class I-specific Tsn, the trioxide-reductase ERp57, and the peptide transporter. According to research, class I assembly entails an optimization phase in which the PLC modifies the complex’s peptide cargo (38, 39). Moreover, this selective peptide loading preferentially supports peptides that exhibit a longer off-rate from the established complex. Researchers have shown that Tsn is the central chaperone regulating PLC activity, while CRT and possibly ERp57 provide additional support (39).

Establishing a hierarchical immune response necessitates the presence of PLC, a transitory multisubunit membrane complex in the ER. The PLC coordinates the translocation of peptides into the ER and loads and modifies the MHC-I molecule. Final proofreading completed in the PLC triggers a T-cell response that explicitly targets cancerous or infected cells by methodically releasing stable peptide-MHC-I complexes to the cell surface. Coordinating seven distinct subunits within a solitary macromolecular assembly is necessary to sample various MHC-I allomorphs (40). These subunits include the MHC-I heterodimer, the oxidoreductase ERp57, the chaperones Tsn and CRT, and the TAP (41). Tsn, CRT, ERp57, and MHC-I circle TAP as two ER-resident editing modules with pseudo-symmetric orientations. A network of multivalent chaperones linking the editing modules creates a proofreading mechanism via two lateral binding platforms associated with MHC-I. The P domain of CRT reaches beyond the MHC-I peptide-binding pocket and extends toward ERp57, whereas the lectin-like domain interacts with the MHC-I glycan. This arrangement suggests that Tsn can clamp MHC-I, which might aid peptide editing. A large ER lumenal cavity is accessible via the membrane entry sites of Tsn and MHC-I, which restrict the translocation pathway of TAP. Because of two lateral windows, antigenic peptides are directed to MHC-I. PLC structures obtained at different stages of assembly provide a mechanistic understanding of MHC-I recruitment and release. Researchers clarified the molecular connection between an ER chaperone network and an ABC transporter in MHC-I construction and offered information on when the adaptive immune response begins (28).

The TAP feature has been well-studied (42, 43). TAP identifies the presence of aromatic, hydrophobic, or positively charged terminal amino acids such as phenylalanine, tyrosine, arginine, or leucine at the peptide’s C-terminus (44). The peptide binding is substantially influenced by the first three residues on the N-terminus. It is ideal to have an N-terminal arginine and aromatic and hydrophobic side chains (45). The C-terminal residue of proteasomes is not very particular, but it does conform to the constraints of MHC-I and TAP molecules (46). Peptides that TAP has translocated must undergo further processing in the ER before binding to MHC-I. The intraluminal processing of peptides is the responsibility of ER aminopeptidase 1 (ERAP1) and ERAP2, which are luminal components of the ER (47). To make peptides that can fit into the MHC-I binding groove, ERAP1 trims amino acids of the N-terminus. These peptides may be 8-11mers long. The structural changes in peptides of this size render ERAP1 incapable of cutting any more (15, 48–50).

The structure of the PLC editing module was revealed by Alexander Domnick et al. (51), who used cryogenic electron microscopy at a resolution of 3.7 Å to determine the PLC’s multi-chaperone-client interaction network. Interactions between multivalent chaperones, including the CRT-engulfed mono-glucosylated MHC-I glycan, and peptide-receptive MHC-I molecules stabilize them, as shown in conjunction with epitope-proofreading studies of the PLC in a near-native lipid environment. After suitable epitopes have been loaded, this glycan can only be processed by α-glucosidase II. Scientists have shown that glycan processing and pMHC-I assembly interact allosterically. This example of interprocess communication exemplifies the well-planned steps in ER quality control and determines when an adaptive immune response starts (51).

The PLC is essential for the effective immune identification of virally and malignantly altered cells because it plays a crucial role in Ag processing. Researchers outlined the relationship between Tsn and MHC-I molecules. Peptide editing was seen in real time by researchers after ultrafast photoconversion to pseudoempty MHC-I molecules. Tsn distinguishes between MHC-I bound to suboptimal cargo and MHC-I loaded with optimum payload. Grasping the kinetics of epitope proofreading requires a thorough understanding of this unique interaction. Researchers utilized all-atom molecular dynamics (MDs) simulations to elucidate the Tsn/MHC-I complex, providing insights into the underlying processes at the atomic level. When cargo is deficient, Tsn’s interaction with MHC-I leads to a reconfiguration of the energy landscape that favors MHC-I complexes with immunodominant epitopes, according to a catalytic working cycle outlined by researchers (52).

This large membrane-bound protein complex was recently crystallized using low-resolution cryo-electron microscopy (cryo-EM), and its atomistic model was based on these data. In an explicit lipid bilayer and water environment, with a total of 1.6 million atoms, the model states that researchers investigated the conformational dynamics of the PLC on a time scale of multimicroseconds using all-atom MDs simulations. An inert, catalytically active core surrounds a bendable protein belt created by two editing modules in the PLC’s layered structure. The PLC’s function involves Tns maintaining the MHC-I binding groove in a conformation similar to that of antigen-loaded MHC-I. The binding groove is approached via a Tns loop in peptide editing led by the MHC-I-linked glycan. Additionally, researchers revealed that CRT affects the dynamics of Tns confirmation via a conformational selection mechanism that helps attract MHC-I into the complex (53).

**Figure 1:**
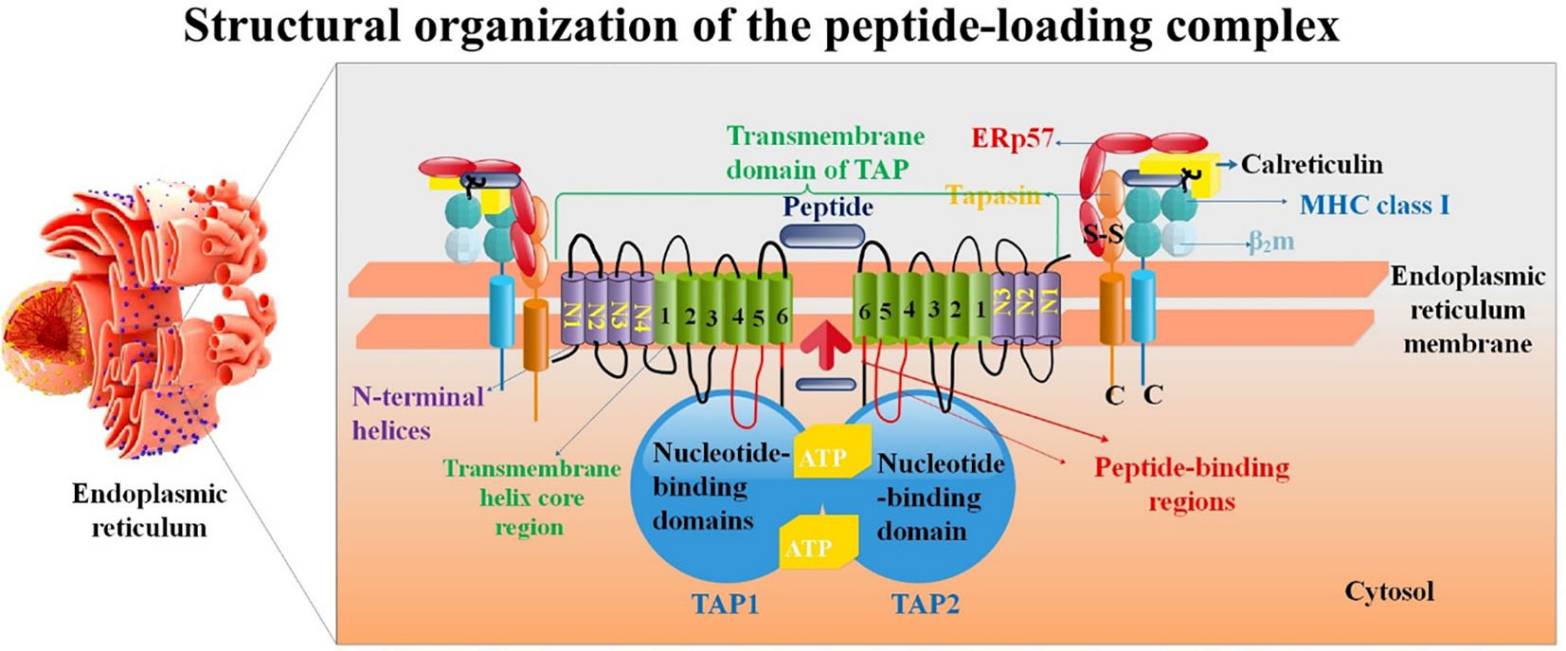
Structure of the PLC. Proteins such as the TAP1/TAP2 complex, ERp57, ER-resident aminopeptidase ERAP1/2, β_2_m, calreticulin, tapasin, and the MHC-I heavy chain are all part of the PLC. TAP has a translocation pore-aligning 6 + 6 TM core domain (H1-H6). Antigenic peptide transport is powered by ATP binding and hydrolysis, which is carried out by Walker A (A), Walker B (B), and C-loop (C) of the nucleotide-binding domain (NBD). It is believed that ATP binding/hydrolysis and peptide transport are connected via an interaction between the L-loop and the Q-loop (55).

The process is contingent upon the PLC, which enables the movement of peptides from the cytosol to the ER and the loading and verification of pMHC-I complexes. The significance of specific PLC components on the pMHC-I complexes displayed remains inadequately comprehended. Researchers utilized stoichiometrically defined antibody–nanobody complexes to quantify distinct MHC-I allomorphs and generated soluble T-cell receptors (sTCRs) to characterize pMHC-I complexes. The researchers identified that different PLC components impacted the pMHC-I surface pool (29, 34, 54). The MHC-I surface composition was altered by knockouts of Tsn, ERp57, or CRT, three PLC editing module components. Researchers demonstrated that an increased ratio of HLA-B*40:01 molecules compensated for the decreased proportion of HLA-A*02:01 presentation. These mutants improved the display of HLA-A*02:01 complexes with inadequate loading and high-affinity peptides overexpressed in the cytosol. According to researchers, the PLC editing module’s components have two functions: they restrict the amount of abundant peptides and serve as peptide proofreaders. This dual purpose guarantees a wide range of antigenic peptides are presented (29) (**Figure 1**).

### 2.1 Endoplasmic reticulum resident protein 57

The core of the MHC-I PLC is formed by the endoplasmic reticulum (ER)-resident proteins TAP, Tsn, and ERp57, which are essential for peptide loading by MHC-I-β_2_m dimers. The PLC is home to a stable heterodimer formed by disulfide connections between ERp57 and Tsn (56). Since ERp57 is involved in the formation of the heavy chain (HC) of MHC-I molecules, it has mainly been investigated for its function in the immune system (57). Enzymes such as phospholipase C, carnitine palmitoyl transferase, oxidase, and reductase rely on it, and it also helps ensure that freshly produced glycoproteins are of high quality in the ER (58).

Additionally, some ER-resident redox protein cofactors, such as protein disulfide isomerase (PDI) and its homologs ERp5, ERp57, and ERp72, are secreted (59, 60). In its role as a 1,25-dihydroxy vitamin D3 (1,25(OH)2D3) receptor, ERp57 regulates several processes, including the quality of newly synthesized glycoproteins, the assembly of MHC-I molecules, immune responses, the unfolded protein response (UPR), the NF-κB and STAT3 pathways, DNA repair, and cytoskeletal remodeling. It also controls immunogenic cell death (ICD) (61).

New evidence suggests that ERp57 is overexpressed in several human cancers (62–64). Additionally, abnormal ERp57 functioning and expression are linked to alterations in the chemosensitivity of tumors and their development and progression. To counteract the chemoresistance and growth of cancer, ERp57 may be a useful biomarker and therapeutic target (62). Quality control, steroid and phospholipid production, post-translational modification of newly generated proteins, Ca^2+^ homeostasis maintenance, and stress response modulation are just a few of the ER’s vital functions within the cell. The ER is populated with numerous proteins to assist in completing these innumerable duties. A thiol oxidoreductase exclusive to ER luminal glycoproteins, ERp57 is a PDI family member. Some of the names given to ERp57 include Grp58, ERp60, ERp61, PDI-Q2, Pdia3, and 1,25D3-MARRS (Membrane Associated, Rapid Response Steroid binding) (57).

The aggregation phenomenon is recognized in ERp57-deficient loading complexes, resulting from the expression of a Tsn mutant (C95A) within a Tsn-negative cell line. This mutant cannot establish a disulfide bond with ERp57. Researchers utilized cell lines that reliably expressed fluorescently tagged Tsn (wild type (WT) and C95A mutant) along with TAP1 to investigate the core loading complex’s assembly, stability, and aggregation. Researchers have indicated that the stability of core loading complexes depends on ERp57. Without ERp57, the core loading complexes will likely create stable aggregates within the ER (56).

Scientists discovered a temporary link between the loading complex and MHC-I molecules in ERp57-deficient cells. Hence, there were almost no MHC-I molecules in the loading complex in the stable state. The model antigen’s presentation and stability of the surface H-2Kb peptide were reduced. Though it does not affect the redox state of MHC-I molecules, researchers showed that ERp57 is a structural component crucial for the stable assembly of the PLC (65).

The 48 kDa glycoprotein known as Tsn was first shown to link the TAP heterodimer and peptide-responsive class I heterodimers. TAP and Tsn are the only accessory molecules and chaperones explicitly implicated in the class I assembly route (66). Recent investigations have demonstrated the necessity of covalent connections between Tsn and ERp57. Tsn forms a somewhat persistent disulfide bond with ERp57, which traps a significant quantity of cellular ERp57 in the PLC, in contrast to many other related chaperones. Recent research suggested that the functional unit *in vivo* responsible for peptide editing is the covalent ERp57–Tsn heterodimer (67).

#### 2.1.1 ERp57 in cancer

Extracellular matrix (ECM) breakdown is necessary for tumor development and invasiveness, and the Src triggers it also drives the polypeptide N-acetylgalactosaminyltransferase (GALNT) activation (GALA) pathway, which causes protein O-glycosylation in the ER. Metalloproteases are needed for ECM breakdown; however, whether other enzymes are also required is not apparent. Studies reveal that in both liver and BC, GALA causes the ER-resident calnexin (Cnx) to become glycosylated (68–70). Because MMP14’s proteolytic activity is reliant on the addition of GalNAc, glycosylation of MMP14 is one mechanism by which GALA increases invasiveness in breast and liver cancer cells (68). ECM degradation sites known as invadosomes are the destinations of trafficked, glycosylated Cnx and its companion, ERp57. Disulfide bridges are shown to be prevalent in the connective and hepatic ECM (71). ECM breakdown requires the reduction of these extracellular disulfide bonds by cell surface Cnx–ERp57 complexes. Hepatocytes do not exhibit cell surface Cnx, although liver cancer cells do *in vivo.* Anti-Cnx antibodies block the formation of liver tumors and the lung metastasis of breast and liver cancer cells. Researchers revealed a Cnx-ERp57 side role at the cell surface that is essential for the formation of tumors and the disintegration of the extracellular matrix (71).

Salvia miltiorrhiza, or Dan Shen, is a famous traditional Chinese herb used to treat BC and many other diseases. That being said, little is known about its mechanics. Salvia miltiorrhiza extract (SME) contains the active component dihydrotanshinone I (DHT), which binds ERp57. DHT may have reduced ERp57 expression in both the RNA and protein forms and ERp57 function. A molecular docking simulation shows that DHT and the ERp57 catalytic site could form a hydrogen bond. Furthermore, in MDA-MB-231 cells, ERp57 overexpression reduced DHT-induced cytotoxicity. Following that, scientists looked into the signaling chain that ERp57 is downstream of. According to the molecular analysis, DHT therapy activated the UPR, ER stress, and cellular death. The researchers suggested that DHT inhibited ERp57, caused ER stress, and activated UPR, all leading to the death of BC cells (72).

One possible biomarker for the early identification of Hepatocellular carcinoma (HCC) is ERp57 expression, which varies considerably across HCC patients, at-risk patients, and healthy persons (73). The overexpression of ERp57 in HCC, colorectal cancer (CRC) (74), and BC (75) has been linked to carcinogenesis and cancer development. ERp57 expression is also connected with cancer cells’ ability to metastasize. High expression of ERp57 is associated with poor overall survival (OS) and high recurrence-free survival rates in adenocarcinoma patients, and it is overexpressed in 73% of cervical malignancies, particularly adenocarcinoma. Cancer metastasis and invasiveness are inhibited in HeLa cells when ERp57 is knocked down *in vitro* (62, 76, 77). There is a correlation between ERp57 levels and prostate cancer progression to the malignant stages (62, 78).

### 2.2 Tapasin in cancer

In the past two decades, the role of Tsn, commonly called TAP binding protein, has undergone thorough investigation and detailed characterization. Tsn plays multiple roles in peptide loading, such as stabilizing TAP, bridging peptide-receptive MHC-I to the TAP transporters, maintaining MHC-I in a peptide-receptive conformation, and facilitating peptide editing, which involves replacing low-affinity cargo with higher-affinity peptides. The implications of Tsn’s role in immune response, especially regarding antigen presentation and CD8+ T-cell differentiation, underscore its significance (79). The exact outcome of MHC-I molecules following peptide loading remains unclear. Peptide-loaded MHC-I molecules do not undergo immediate export from the ER following their dissociation from TAP, indicating that additional regulatory inspections and sorting processes occur after the PLC. UDP-glucose is a critical component in the post-PLC quality control process, specifically through the action of UDP-glucuronosyltransferases 1 (UGT 1), an enzyme located in the ER/cis-Golgi responsible for overseeing the proper folding of glycoproteins (80). By reglucosylating MHC-I molecules linked to less-than-ideal ligands, UGT1 can make CRT recognize these molecules again. This enables them to reconnect with the PLC and has been shown to affect the best possible peptide choice (81).

Loss of Tsn helps malignancies evade the immune system and diminish their immunogenicity (82). It was first shown *in vivo* in mice tumor models how crucial Tsn is for antigen presentation in cancer. An LC cell line that downregulated many MHC I pathway components was implanted into mice. Against this backdrop, Tsn transfection was enough to improve antigen presentation, boost the immune response specific to the antigen, slow tumor development, and improve survival (83). Researchers determined whether there is a relationship between Tsn expression and the presence of CD8+ cytotoxic T lymphocytes (CTLs) in CRC, as well as OS. Scientists have discovered that Tsn aids in antigen presentation, tumor immune detection, and CD8+ CTL destruction; hence, a decrease in Tsn expression is linked to tumor growth in CRC (84).

Shionoya et al. (85) found a favorable correlation between Tsn expression and patient survival in their study of 85 primary tumor lesions from NSCLC patients. CD8+ T-cell infiltration of tumor lesions was shown to be favorably linked with survival and to have a synergistic effect with *Tsn* expression. Researchers used the CRISPR/Cas9 technology to specifically target the *Tsn* gene to prove a causal relationship between Tsn deletion and CTL recognition in cancer models derived from humans. Their goal was to create human lung and colon cancer cells lacking in Tsn. To test the efficacy of each Tsn-proficient WT, researchers stimulated CTLs that recognize endogenous tumor-associated antigens (TAA), survivin, or cep55. However, despite expressing the antigen, neither CTL line paid any attention to the Tsn-deficient mutants. Also, animals with the Tsn-deficient version still developed tumors even after receiving the cep55-specific CTL line by adoptive transfer. The inability to process TAA antigens due to Tsn loss probably allowed the escape from CTL identification that was unique to TAAs. Expression of Tsn is, consequently, crucial for CTL monitoring of human malignancies (85).

The three oral squamous cell carcinoma (OSCC) cell lines that were studied showed evidence of Tsn promoter methylation. Additionally, the levels of Tsn mRNA and protein were shown to rise significantly after treatment with 5-aza-2′-deoxycytidine. Potentially useful as a predictive biomarker, the downregulation of Tsn is linked to worse clinical outcomes in individuals with OSCC. The downregulation of Tsn in OSCC may be influenced by promoter methylation (86).

Immune recognition and antigen presentation depend on the catalytic chaperone Tsn in the PLC loading MHC-I molecules with peptides. A significant limitation to mechanistic understanding has been the absence of comprehensive structural data for Tsn–MHC–I. Researchers revealed crystal structures of human Tsn complexed with both anti-Tsn antibodies and the MHC-I protein HLA-B*44:05. HLA-B*44:05’s Tsn-stabilized peptide-receptive state is characterized by peptide binding groove distortion and β_2_m interaction instability, resulting in peptide release. The migration of the membrane-proximal immunoglobulin-like domains of Tsn, HLA-B*44:05, and β_2_M synchronizes with the change to a peptide-receptive state. Combined, these crystal structures provide light on a unique mechanism of Tsn-mediated peptide exchange (87).

### 2.3 MHC-I peptide processing and presentation

The process of antigen presentation through MHC-I molecules represents a complex and essential protective mechanism employed by the adaptive immune system to combat pathogens and malignant cells (88). Part of the superfamily of ATP-binding cassette (ABC) proteins, the heterodimeric TAP is an essential component of the antigen presentation pathway. A nucleotide-binding domain is connected to six transmembrane helices in the core transporter region of the TAP subunits TAP1 and TAP2 (ABCB2 and ABCB3, respectively). Additionally, each core subunit is N-terminally attached to TMD0, an additional transmembrane domain with four helices (89). TAP is an integral part of the ER-resident PLC because it serves as a gatekeeper for the reservoir of antigenic peptides created by cytosolic proteasomal degradation. TAP carries peptides with overlapping lengths and sequence specificities to engage with MHC-I molecules and ensure MHC-I antigen presentation. The MHC-I peptide-binding pocket is aligned with the aminopeptidases ERAP1/2 because they can digest longer peptides in the ER lumen. Tsn carries out peptide proofreading, whereas peptide loading is aided by the chaperones CRT and ERp57, which guarantee the proper assembly of MHC-I molecules and the editing module (90–92). Researchers found a way to control antigen presentation by adding a photo-caged amino acid to TAP’s catalytic ATP-binding domain, as our knowledge of MHC-I trafficking pathways is still restricted. If researchers can start TAP-dependent antigen translocation by optical manipulation, they may learn more about TAP’s function in live cell PLC and MHC-I trafficking. This adaptable approach can be used to investigate other cellular pathways that are affected by P-loop ATP/GTPases (92).

Dendritic cells (DCs) present antigenic peptides on MHC molecules to T-cells, thereby orchestrating immunological responses. The fundamental component of the supramolecular structure referred to as the PLC, essential for antigen processing and presentation through MHC-I, is the peptide transporter located in the ER membrane, commonly known as TAP (93, 94). According to the research, the PLC attracts extra proteins such as B-cell receptor-associated protein 31, vesicle-associated membrane protein-associated protein A (VAPA), and extended synaptotagmin-1 (ESYT1) as DC develops and matures. Scientists have found the proteins that bind to specific sites and export cargo from the ER to nearby TAP, which is located 40 nm from the PLC. Based on these findings, the APM and the locations of membrane contact and ER departure are closely related. The functions of BAP31, VAPA, and ESYT1 were similar within the framework of DC MHC-I antigen processing. Nevertheless, the examination of single-gene deletions of the recognized PLC interaction partners revealed that the MHC-I surface expression was considerably decreased when TAP and Tsn were deleted using CRISPR/Cas9 technology. Researchers demonstrated the dynamic and adaptable nature of the PLC composition in DCs, an aspect not identified through cell line studies (32).

Furthermore, researchers investigate the role of the chaperone Tsn-binding protein-related (TAPBPR) in facilitating peptide loading. According to the researchers, TAPBPR attaches to the F pocket and allosterically changes the structures of the distant pocket B to produce a peptide-receptive conformation ideal for tolerating the arriving N-terminus. Researchers elucidated the molecular mechanisms involved in peptide loading onto MHC-I, both in the presence and absence of chaperones (95).

Also, scientists showed that the binding of TAPBPR at the MHC-I pocket-F may facilitate the N-terminal loading of the following peptide by considerably influencing the distant pocket-B via allosteric modulation. Researchers demonstrated that the partly loaded peptide has the potential to significantly decrease the stability of the TAPBPR-MHC complex by inducing the SL (TAPBPR scoop-loop) region to detach from the pocket-F and adopt a more solvent-exposed conformation. Additional structural investigations indicate that the peptide loading may indirectly use direct contacts and allosteric perturbations to affect the SL binding site. Moreover, it was discovered that the jack hairpin region, another structural feature of TAPBPR, assisted in facilitating peptide editing. Researchers identified the critical structural elements that control the kinetics of peptide loading and provide insight into the intricate molecular processes behind the process of peptide loading into TAPBPR-bound MHC-I (96).

Polymorphisms in the *endoplasmic reticulum aminopeptidase* (*ERAP*) genes, namely *ERAP1* and *ERAP2*, have recently been shown to be much more prevalent in individuals with ankylosing spondylitis (AS), psoriasis, or Behçet’s disease (BD), according to genetic association studies. The fact that ERAP is in epistasis with the risk alleles of the MHC-I allele is significant under these settings. Nevertheless, it has come to light that not all AS or BD patients possess the MHC-I risk allele. This finding has sparked debates over the significance of this commonality and whether it indicates a common illness origin (97–99) Jonas J W Kuiper et al. mapped the *ERAP1* and ERAP2 haplotypes in 84 cases and 890 controls from the Netherlands (100). A relationship was found at variation rs10044354, which led to a significant upregulation of ERAP2 expression, according to the researchers. Separately, researchers found and cloned an ERAP1 haplotype (tagged by rs2287987) that was related to over 50% of the cases; this ERAP1 haplotype serves as both a risk factor and a protective factor for additional MHC-I-opathies. Based on transcriptome data (n = 360), researchers found that the risk ERAP1 haplotype caused a dramatic change in ERAP1 isoform expression, leading to reduced protein expression and unique enzymatic activity. A separate Spanish cohort consisting of 46 cases and 2,103 controls confirmed and confirmed the direction of impact for both rs10044354 [meta-analysis: odds ratio (OR) [95% CI]=2.07[1.58-2.71], P = 1.24 × 10(−7)] and rs2287987 [OR[95% CI]: =2.01[1.51-2.67], P = 1.41 × 10(−6)]. The rs2287987-rs10044354 haplotype was more strongly related to Birdshot in both cohorts compared to the individual variants. At last, after analyzing three European populations (n = 3353), researchers found that the ERAP1 background influences the expression of the ERAP2 protein. Lastly, Birdshot is characterized by a functionally different mix of ERAP1 and ERAP2, which supports the idea that techniques may be developed to rectify ERAP function in order to cure Birdshot and other MHC-I-opathies (100).

#### 2.3.1 MHC I in cancer

Numerous innate and adaptive immune cells, such as DCs, macrophages, neutrophils, natural killer cells (NK), γδ T-cells, CD4+ T-cells, and CD8+ T-cells (cytotoxic T-cells), may infiltrate solid tumors. Some of these immune cells can even eliminate particular tumors. Tumor cells may effectively evade the immune system’s regulation by activating several internal processes and modifying external variables in return. It is now thought that immune escape is one of many tactics cancers use to protect their survival and growth—also referred to as the cancer hallmarks. These molecular immune escape mechanisms involve the following: A) obstructing MHC-mediated antigen presentation to hinder T-cell surveillance; B) releasing broadly immunosuppressive cytokines like TGF-β and IL-10; and C) displaying inhibitory receptors on the cell surface such as PD-L1 and PD-L2, which limit T-cell activation (101).

Using a human melanoma cell line, researchers conducted a genome-wide CRISPR screen. Contrary regulators of MHC-I transcription were the deubiquitinating enzyme BRCA1-Associated Protein 1 (BAP1) and the polycomb repressive complex 1 (PRC1) component Polycomb Group RING Finger Protein 1 (PCGF1). PCGF1 facilitates ubiquitin deposition at H2AK119, which silences the MHC-I promoters. BAP1 reverses this change, restoring MHC-I expression. Despite PCGF1’s extensive-expression in malignancies, certain tumor lines, particularly MHC-I low cancers, demonstrated enhanced MHC-I expression with a decrease in PCGF1. Both PRC1 and PRC2 work together to suppress low transcription in cells with low MHC-I expression. Rest assured, MHC-I expression was amplified, and T-cell-mediated tumor cell killing was reinstated after PCGF1 elimination. Researchers light up an additional mechanism by which malignancies regulate MHC-I expression: epigenetic repression by the PRC1 component PCGF1 (102).

The HLA-B27 subtypes that are related to spondyloarthritis (SpA), namely B*27:02, B*27:05, and B*27:07, have a higher propensity to aggregate in cytoplasmic vesicles and form intracellular oligomers compared to the non-SpA-associated subtypes HLA-B*07:02 and HLA-B*27:06. Better to understand the relationship between SpA propensity and HLA-B-containing vesicles, researchers set out to describe their composition and nature. Subtypes of HLA-B27 prone to SpA have an accumulation of misfolded HLA-B HC, β_2_m, and ER chaperones from vesicles produced from the ER that are distinct from the PLC. Through a noncanonical route, this behavior may contribute to the pathogenicity of HLA-B27 (103).

Peptides containing proline at position 2 bind preferentially to MHC-I molecules of the HLA-B7 supertype. Two subpeptidomes are found in HLA-B*51:01 and B*51:08, with one subpeptidome enriched at position 1 with Ala2 and Asp and the other with Pro2 and hydrophobic residues at P1. To explore the existence of subpeptidomes across various allotypes, researchers provided a meta-analysis of the peptidomes offered by molecules of the B7 supertype. The presence or absence of a Pro or other residue at position 2 differentiated the subpeptidomes of many allotypes. Glu1 was present in Ala2-containing ligands, whereas Asp1 was chosen by the Ala2 subpeptidomes in HLA-B*54:01. Researchers suggested that subpeptidomes may be present at MHC HC sites 45 and 67 based on sequence alignment and crystal structure analysis. Antigen presentation in other MHC-I molecules may be better understood if the principles behind the existence of subpeptidomes can be deciphered—efficacy of HLA-B7 supertype subpeptidome selection (104).

Due to gene loss or epigenetic silencing, cancers may lose the production of non-essential molecules and are often genetically unstable. Molecules belonging to the MHC-I antigen presentation pathway, including MHC-I, are not essential for cell survival and proliferation. Consequently, tumors may inhibit or eliminate MHC-I antigen presentation, rendering them less stimulatory or invisible to CD8 T-cells without compromising tumor growth and metastasis (88). The researchers examined the therapeutic implications, mode of action, and frequency of MHC-I deletion in cancers. Because of their clinical significance, this overview mainly focuses on human malignancies, except those mentioned. Cancers may also avoid immune clearance by expressing HLA-E and HLA-G, two “non-classical” MHC-Ib molecules. Studies examining MHC-I expression in primary patient samples have mainly used immunohistochemistry (IHC). Traditional MHC-I molecules, including HLA-A, HLA-B, and HLA-C, and monomorphic determinants on their HC may be targeted by antibodies to perform IHC. This has led to a downregulation of MHC-I antigen expression in several cancers. There have also been reports of a single MHC-I molecule losing its expression. Many investigations have shown MHC-I negative malignancies; however, due to IHC’s sensitivity limitations, some patients could display some MHC-I molecules (88).

Along with HLA-F and HLA-H, HLA-G and HLA-E are members of the non-classical HLA-class Ib family. Numerous studies have examined these molecules’ function in regulating the immune response in both healthy and diseased settings. Different immune cell types, including T-cells, antigen-presenting cells, and immunoregulatory cell populations, including mesenchymal stem cells, may also express HLA-G. However, HLA-G is up-regulated in a variety of clinical circumstances, including inflammatory disorders, malignancies, viral infections, and transplantation. Transformed cells (tumor and virus-infected cells) that express HLA-G have an immune escape mechanism that prevents them from being recognized and lysed by cytotoxic immune effectors such as NK cells and cytotoxic T lymphocytes (105–107).

Furthermore, HLA-E binds peptides isolated from the leader sequence of HLA-class I molecules (HLA-A, -B, -C, and -G) and delivers them to NK cells via the inhibitory receptor CD94/NKG2A. This prevents NK cell lysis of cells expressing normal amounts of HLA-class I molecules. When HLA-class I expression is low, cells produce fewer HLA-I-derived peptides, which means they have less HLA-E and are more susceptible to NK cell lysis. When HLA-E binds to peptides produced by HLA-G, it may cause an interaction with the CD94/NKG2C activating receptor on NK cells. When HLA-G+ trophoblast cells invade the placenta, this property is used to trigger NK cell lysis, which in turn causes tissue remodeling (106). Furthermore, Researchers showed that mesenchymal stromal cells produced from gestational tissue, namely from cord blood, are not very immunogenic. This is associated with the fact that these cells co-express HLA-G and HLA-E (106, 108). DCs release exosomes (EXOs), which carry antigens and include MHC-I and MHC-II, along with other components. Immunogenicity of EXOs MHC-I has been shown by scientific studies when these molecules are “indirectly” loaded onto DC cells using peptide addition. A peptide binding study was not carried out to establish a relationship between the quantity of MHC-I/peptide complexes on the EXOs and the T-cell-stimulating function. Scientists tested the EXOs’ activation potencies in T-cell activation assays and analyzed peptide binding to MHC-I under different loading conditions (109–112).

**Figure 2:**
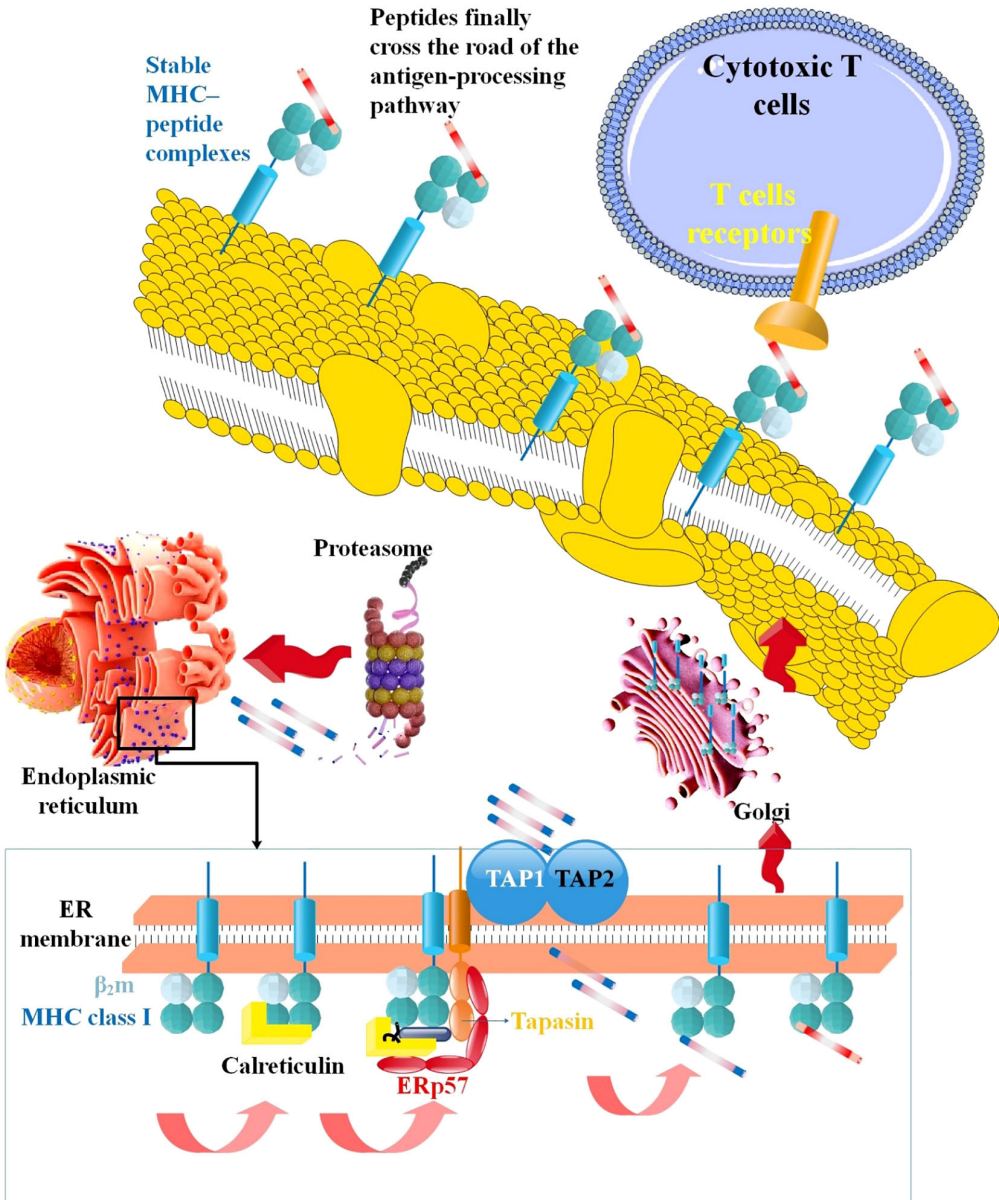
The mechanism via which MHC-I processes antigens. TAP carries them into the ER lumen to load antigenic peptides produced by the proteasome in the cytosol onto MHC-I, and TAP carries them into the ER lumen. CTL recognize stable MHC-peptide complexes when they exit ER via the Golgi network and reach the cell surface (115).

Researchers showed that peptides may be directly and much more strongly loaded onto pure EXOs’ MHC-I than by indirect loading. Even without exogenous β_2_m, the moderately acidic circumstances in which the direct loading approach was carried out proved efficient. EXO potency was significantly increased by this increase in peptide binding, enabling researchers to investigate EXO biologic activity *in vitro.* When combined with APC, EXOs carrying the HLA-A2/MART1 tumor peptide stimulated a T-cell line targeting HLA-A2/MART1. Using HLA-A2neg APC, the T-cells reacted to EXOs, indicating that functional MHC-I/peptide complexes were transferred to APC rather than peptides alone. Under the same circumstances as MHC-I, MHC-II molecules—highly expressed on DC EXOs—were likewise functionally loaded. This property facilitates the transmission of a wide range of peptide antigens capable of activating CD4+ T helper cells and CD8+ cytotoxic T-cells, both essential for an effective antitumor response. The ideal loading conditions and capacity for transmitting both MHC-I and MHC-II antigens to antigen-presenting cells have resulted in the advancement of extracellular vesicles as a “acellular” immunotherapy approach, which is presently under investigation in clinical trials (113, 114) (**Figure 2**).

#### 2.3.2 HLA associated with cancer

CTLs are exposed to antigenic peptides via HLA-I. An essential phase in the creation of CTL responses is this one. The degree of HLA-I expression directly influences NK-cell responses, although peptide-HLA-I (pHLA-I) complex quantity and quality are also crucial for CTL responses. Antigen processing machinery (APM) proteins have a role in choosing which peptides are given and in the maturation of HLA-I. These proteins thus have a significant role in determining how the immune system reacts to cells in both health and illness. Scholars specifically focus on the multifunctional protein Tsn, which is peculiar to HLA-I. In light of the varying Tsn-dependencies across HLA-I allomorphs, researchers also examine the unique characteristics of each allomorph in terms of development, structure, and association with malignant illnesses, namely brain tumors (116).

Autologous treatments are hindered by polymorphic and unstable MHC-I and MHC-like molecules with inadequate peptides, metabolites, and glycolipids. These features complicate antigen-specific TCR and disease-relevant antigen identification (117). To bind to the MHC-I, researchers linked conserved epitopes across the HC/β_2_m interface using an engineered disulfide linkage, therefore enabling positive allosteric interaction between the peptide and light chain (β_2_m) subunits. These let researchers create “open MHC-I,” conformationally stable peptide-receptive molecules (117).

Furthermore, loaded with low- to moderate-affinity peptides, open MHC-I molecules are well-folded and more thermally stable than WT. Researchers investigated how the disulfide link influences MHC-I kinetics and conformation by use of solution Nuclear Magnetic Resonance (NMR). Observed were long-term effects on the α2-1 helix and α3 domain as well as local modifications in the β_2_m-interacting sections of the peptide-binding groove. The interchain disulfide bond stabilizes MHC-I molecules in an open conformation, therefore enabling peptide interchange across five HLA-A supertypes, six HLA-B supertypes, and oligomorphic HLA-Ib molecules. Stable and ready-to-load MHC-I systems may be built by researchers using structure-guided design and conditional β-peptide ligands. This enables highly polymorphic HLA-I allotypes and oligomorphic nonclassical molecules to screen libraries of antigenic epitopes and polyclonal TCR repertoires (117).

Researchers investigated the possibility that peptides directly associated with β_2_m may form a complex with the human HLA-I HC and be identified as components of the cell surface and soluble reagents by human CTL. The N terminus of human β_2_m was physically associated with a peptide epitope limited by HLA-A2. When this fusion protein was multimerized, it formed complexes called “fusamers” that linked exclusively to specific CTL clones. The HLA-A2 HC also refolded quickly in a laboratory setting. These fused peptide/MHC complexes were equally effective as traditional tetrameric peptide/MHC complexes in detecting CTL specific to antigens. After delivering the fusion protein via a retroviral vector, appropriate CTL clones identified and eliminated the target cells. β_2_m-negative, TAP-negative and unmutated B cell lines showed adequate sensitization to CTL lysis, suggesting these constructs might help trigger CTL even when the MHC-I pathway is disrupted. Retroviral vectors carrying specific peptides covalently bound to β2m might be helpful for *in vivo* CTL priming against therapeutically relevant epitopes (118).

When CTL detects pMHC-I complexes on target cells, they lyse them. The lack of Tsn, a critical component of the PLC, impacts the surface repertoire of MHC-I peptides. Analysis of 85 primary tumor lesions from non-small cell lung cancer (NSCLC) patients revealed a statistically significant association between Tsn expression and OS. Together with Tsn expression, CD8+ T-cell infiltration of tumor lesions was seen as positively linked with survival. Researchers used the CRISPR/Cas9 technology to target the *Tsn* gene, resulting in Tsn-deficient human lung and colon cancer cells. This was done to demonstrate that in human cancer models, there is a clear association between the loss of Tsn and CTL recognition. The researchers primed the CTLs to respond to endogenous tumor-associated antigens (TAA), survivin, or cep55, and each Tsn-proficient WT. On the other hand, even though the Tsn-deficient mutants expressed antigen, both CTL lines disregarded them. Furthermore, in mice with the Tsn-deficient version, adoptive transfer of the cep55-specific CTL line could not stop tumor development. Tsn loss most likely restricted TAA antigen processing and allowed escape from CTL recognition specific to TAAs. To protect human malignancies from CTL surveillance, Tsn expression is crucial (85).

Tumor cell death after CTL recognition of TAAs on tumor cells is thought to be directly correlated with TAA protein production and reliant on the amount of peptide present in the HLA-I molecule’s binding site (117, 119). On human tumor cell lines, researchers looked for evidence of a connection between the expression levels of the Her-2/neu protein and the presentation and recognition of CTL by the HLA-A*0201/Her2/neu peptide. Scientists developed a TCR mimic (TCRm) monoclonal antibody (mAb) called 1B8 to locate the Her2_(369)_-A2 complex, which is represented by the HLA-A2.1/Her2/neu peptide_(369-377)_ (117). A possible explanation for the observed quantitative differences in the Her2_(369)_-A2 complex levels on the five human tumor cell lines studied is the variable degree of TCRm mAb labeling. No significant correlation was seen between the levels of Her2/neu Ag, HLA-A2 molecule, and Her2_(369)_-A2 complex expression in tumor cell lines that were pretreated with IFN-γ and TNF-α for Her2/neu protein and HLA-A2 molecule expression. Additionally, there was a clear correlation between the enhanced tumor cell mortality and the elevation in Her2_(369)_-A2 epitope density in cytokine-treated cell lines compared to untreated cells. Her2_(369)_-A2 complex level for untreated cells showed a trend with tumor cell lysis, although the relationship was not statistically significant. These results imply that rather than the overall amount of TAA expression, the vulnerability of tumor cells to CTL-mediated lysis may be anticipated based on the degree of particular pMHC-I expression. These investigations also demonstrated the potential of the TCRm mAb for validating endogenous HLA-peptide epitopes on tumor cells (120).

**Figure 3:**
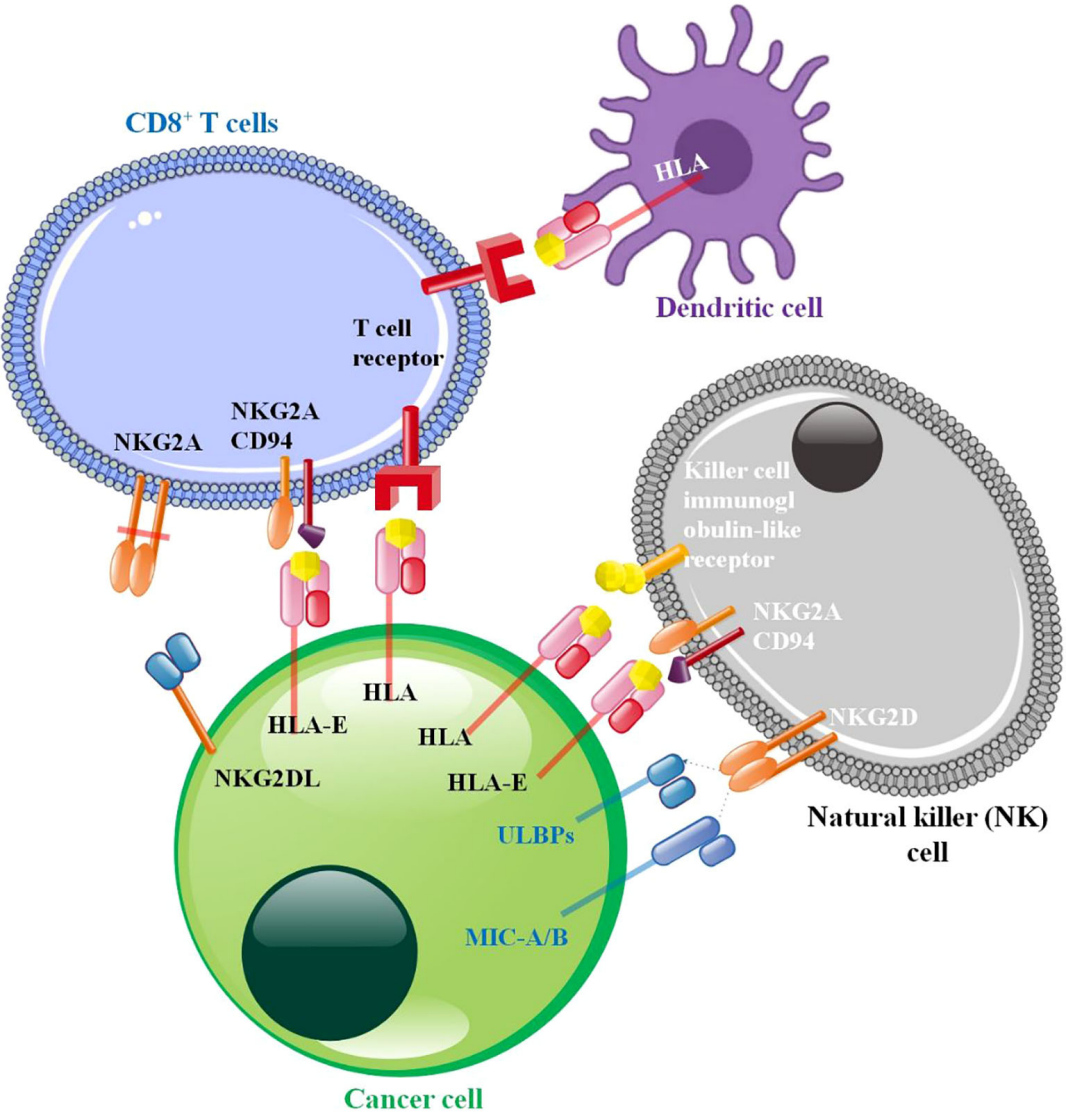
Cancer cell-immune cell interaction in tumor microenvironment. The NK cell illustrates well. NK cell surface immunoglobulin-like receptor (KIR), a conventional MHC-I inhibitory receptor. NK cell activation is inhibited by KIR and MHC-I molecules in normal cells. Their findings suggest that HLA-E: CD94-NKG2A blockage may cure cancer. HLA-G suppresses NK cell function by binding to its receptors ILT2 and KIR2DL4. The best-studied activating receptor is NKG2D, which recognizes MIC and various UL16-binding proteins (ULBPs). Continuous activating receptor activation on NK cells promotes desensitization. NK cell desensitization occurs when tumor cell ligands MHC class I chain-related protein A (MICA) and MICB directly interact with NKG2D. The anti-cancer response may be reduced by blocking TCR-MHC-I interaction. T and NK cells have an inhibitory receptor formed when NKG2A and CD94 dimerize. T-cells produce an inhibitory signal when they come into contact with peptide-loading HLA-E, which phosphorylates ITIM. Enhancement of NKG2A and HLA-E by cancer cells (122–126).

Mutations in the *p53* gene typically result in an excess of the WT p53 protein, representing the most common genetic alterations observed in human cancers. The potential for T lymphocytes to react to tumor cells that overexpress WT or mutant p53-derived peptides supports the application of these epitopes in cancer immunotherapies. The researchers employed two separate flow cytometry-based assay techniques to assess the binding capabilities of WT and mutant p53 peptides to HLA-A2.1: the T2 MHC-I peptide stabilization assay and the peptide-induced MHC-I reconstitution test. The reason is that antigen-specific T-cell recognition requires peptide binding to MHC-I molecules. Twenty WT sequences were selected because they all met the criteria for the HLA-A2.1 peptide-binding motif. From the WT peptides attached to HLA-A2.1 that were previously chosen, seven WT p53 and two out of thirteen mutant p53 peptides were generated for the stabilization and reconstitution assays. The observed lower affinity for HLA-A2.1 could account for detecting only six additional WT and six mutant p53 peptides in the reconstitution experiment. Hypothesized to serve as immunogens for the *in vitro* and *in vivo* generation of cytolytic T-cells specific to HLA-A2.1, these p53 peptides bind HLA-A2.1 (121) (**Figure 3**, **Table 1**).

**Table 1:** The function of complicated peptide loading compounds in various cancer types.

Cancer	Peptide-loading complex compounds	Explain	Ref
Liver and breast cancer (BC)	Cnx-ERp57	Researchers discovered a crucial secondary function for Cnx-ERp57 at the cell surface: it plays a role in the degradation of the extracellular matrix and tumor development.	(71)
BC	ERp57	The researchers suggested that DHT inhibited ERp57, caused ER stress, and activated UPR, all of which led to the death of BC cells.	(72)
Colon cancer	ER-resident thiol oxidoreductase ERp57	A decrease in p70S6K phosphorylation suggests that mTORC1 phosphorylation activity was reduced by ERp57 elimination. The research results indicated that ERp57 might be an effective target for cancer treatments.	(127)
BC	ERp57	ERp57 is a multipurpose chaperone that may control various biological processes to preserve BC cells’ homeostasis and encourage the growth of bone metastases.	(128)
Melanoma cancer	MHC-I	MHC-I expression was amplified, and T-cell-mediated tumor cell killing was reinstated after PCGF1 elimination. Researchers light up an additional mechanism by which malignancies regulate MHC-I expression: epigenetic repression by the PRC1 component PCGF1.	(102)
Brain tumors	HLA-I	The degree of HLA-I expression directly influences NK-cell responses, although peptide-HLA-I (pHLA-I) complex quantity and quality are also crucial for CTL responses. Researchers also examine the unique characteristics of each allomorph in terms of development, structure, and association with malignant illnesses, namely brain tumors.	(116)
All type cancer	MHC-peptide	Human CTL can identify peptides directly linked to β_2_m as soluble reagents and components of the cell surface and potential complexes with the human HLA-I HC. Potentially valuable tools for *in vivo* CTL priming against therapeutically important epitopes might be specific peptides covalently bound to β_2_m and delivered utilizing retroviral vectors.	(118)
Non-small cell lung cancer (NSCLC),	pMHC-I	Tsn loss most likely restricted TAA antigen processing and allowed escape from CTL recognition specific to TAAs. Tsn expression is crucial for protecting human malignancies from CTL surveillance.	(85)
The five human tumor cell lines	Peptide-HLA	These findings suggest that the degree of specific pMHC-I expression may be used to predict tumor cells’ susceptibility to CTL-mediated lysis rather than the total quantity of TAA expression.	(120)
Small cell lung cancer (SCLC)	TAP and Tsn	Nevertheless, the TAP-, Tsn-independent peptides were not provided when these tests were expanded to tumor cell lines generated from SCLC, which researchers demonstrated to be Tsn deficient in addition to TAP-negative. Pre-treating SCLC cells with IFN-γ might address this lack of presentation. Or, the TAP-, Tsn-independent peptides were effectively delivered by SCLC cells using an ER signal sequence that guided them into the ER.	(129)
CT-26 colon cancer and the B16-F10 melanoma	Used peptides generated by the influenza virus that were matched with MHC for translation	High efficacies against tumor challenge were found in CT-26 colon cancer and B16-F10 melanoma, indicating the potential general application of this novel vaccine. This idea might treat human tumors using foreign peptide ligands tailored to a particular MHC-I haplotype.	(130)
Modified Wilms’ Tumor 1 (mWT1) protein	HLA-A24	The initial step is forming an encounter complex between the 9-residue mWT1 fragment peptide and the cluster of damaging residues on the surface of HLA-A24. Although the PLC could help stabilize the MHC molecule, the antigen peptide’s inherent affinity for the MHC molecule matters when it comes to binding.	(131)

Both the conserved and invariant c-type lectin CD94/NKG2A inhibitory receptor (henceforth referred to as NKG2A, which stands for NK cell protein Group 2-A) that binds non-classical HLA-E and the polymorphic killer-cell immunoglobulin-like receptors (KIR) that bind classical HLA-I molecules HLA-A, HLA-B, and HLA-C are significant inhibitory receptors that can instruct NK cells. HLA-E requires the input of peptides from classical HLA-A, HLA-B, or HLA-C for correct folding and cell surface trafficking (132, 133). A meta-analysis conducted by Norman Shreeve et al. found that effects such as suboptimal maternal vascular responses during pregnancy, altered placental gene expression, decreased fetal weight, increased rates of smaller fetuses with asymmetric growth, and abnormal brain development were caused by NKG2A genetic ablation in dams mated with wild-type males. These symptoms are seen in pregnant women with pre-eclampsia. A genome-wide association study of 7,219 pre-eclampsia patients found that when the maternal HLA-B allele did not boost NKG2A education, there was a 7% higher relative risk. Researchers’ findings showed that the mother’s HLA-B→HLA-E→NKG2A pathway contributes to a healthy pregnancy and may affect the children’s health, showing the physiological significance of NK cell education (134).

## 3 Peptide loading complex in liquid tumors

The binding of peptides to MHC-I in the ER is enhanced by the presence of PLC, which includes CRT. CRT binds to MHC-I through a conserved glycan, facilitating its movement to the PLC for peptide binding. Somatic frameshift mutations in CRT lead to a specific subgroup of myeloproliferative neoplasms characterized by ongoing blood malignancies, resulting in proliferation (CRT-FS) (135). All CRT-FS proteins exhibit a net negatively charged C-terminal sequence instead of a positively charged one, and they lack the typical ER-retention signal. Najla Arshad and colleagues examined the influence of CRT-FS on antigen presentation via MHC-I in human cells. Research indicates that CRT-FS cannot facilitate the PLC peptide-loading function of CRT. Although CRT-FS expression did not enhance high-affinity peptide binding, the observed reduction in surface MHC-I levels in CRT-deficient cells was associated with a decrease in high-affinity peptide binding. Despite the secretion of CRT-FS and its lack of binding to the PLC, it could still bind to MHC-I glycan independently. This observation explained the suboptimal recruitment of MHC-I. The introduction of the ER-retention sequence to CRT-FS successfully restored its interaction with the PLC; however, it did not enhance surface expression or MHC-I recruitment. This suggests that the mutations in CRT-FS significantly impair the functionality of the PLC. Since tumor cells may avoid immune monitoring when MHC-I is down-regulated, these results could be crucial in developing immunotherapies that effectively manage myeloproliferative neoplasms (136).

New medications have been discovered, and various therapy techniques have been developed in recent years as our understanding of the pathophysiology of acute myeloid leukemia (AML) has advanced (137). The development of AML immunotherapies and their clinical translation has been sluggish. Intrinsic AML characteristics hinder the process of moving from immunobiology to immunotherapy. Genetic/epigenetic heterogeneity and subclonal contribute to biologic variance in AML, a family of distinct cancers rather than a single illness (138, 139). Immunosuppression in the AML microenvironment is linked to global alterations in the profiles of immune cells in the bone marrow. Additionally, the microenvironment shields leukemic stem cells—which may cause relapse—from being destroyed by therapy or the immune system (140).

The absence of specific immune targets is the fundamental reason why novel immunotherapies have failed to improve outcomes for patients with AML. Scientists isolated 58 tumor-specific antigens (TSAs) by using a novel proteogenomic strategy to the immunopeptidome associated with MHC-I in 19 primary AML samples. Most of these TSAs (86%, to be exact) originated from purportedly non-coding genomic areas and did not include any mutations. Intron retention, epigenetic alterations, and TSA biogenesis were all aided by two AML-specific abnormalities. Both blasts and leukemic stem cells exhibited AML TSA-coding transcripts, which were substantially shared across individuals. Improved survival, immunoediting, accumulation of activated cytotoxic T-cells, and spontaneous expansion of cognate TCR clonotypes were all associated with the expected amount of TSAs in AML patients. Immunotherapy against AML could be a promising strategy if it targets these TSAs (141). The discovery of MHC-I-associated peptides originating from any reading frame of any genomic region (not only exons) has been made possible using a proteogenomic strategy that was recently developed. Eighteen primary human AML samples were analyzed, and 82 TSAs were discovered. Even though traditional EXO-based methods would have missed these TSAs, they were all produced from commonly expressed transcripts with mutations. Consistent with earlier findings that increased intron retention is an essential characteristic of AML, researchers discovered that introns were the primary source of TSAs. Approximately 98.04 percent of the global population has one of the 24 TSA-presenting HLA allotypes. While all of them were either expressed at deficient levels or not expressed at all in normal tissues (from the “Genotype-Tissue Expression” (GTEx) project), the majority of them were represented by at least 50% of AML samples in the “The Cancer Genome Atlas” (TCGA) cohort. The precise epigenetic modifications that cause cancer cells to produce TSA remain unknown to researchers. According to the researchers, there is no concern about collateral harm to healthy normal tissues, and immunization against aberrantly expressed TSAs might be utilized to cure most patients (142).

Particular proteins involved in the processing pathway of HLA-I and HLA-II complexes are responsible for antigen presentation. The invariant chain (II) peptide Class II-associated Invariant Chain Peptide (CLIP) is necessary for HLA-II-mediated antigen presentation since it stabilizes HLA-II molecules before antigen loading by transiently and non-specifically attaching to various HLA-II receptor grooves. Scientists proved that CLIP binds to leukemic cells’ surface HLA-I molecules differently. Scientists discovered that CLIP peptide display in HLA-II-negative AML cells (143). A direct involvement of CLIP in the HLA-I antigen presentation pathway was shown when HLA-I cell surface display was diminished in AML cells by silencing Ii. Five Ii-derived peptides, including two from the CLIP region, were detected in HLA-I-specific peptide eluates from B-LCLs. According to peptide binding experiments, the eluted CLIP peptide RMATPLLMQALPM is firmly bound to four different HLA-I supertypes (-A2, -B7, -A3, -B40*) in vitro.* In addition, these four supertypes are bound by shorter versions of this CLIP peptide, even if in silico methods only foretold binding to HLA-A2 or -B7. No CTL responses were induced when these peptides were administered to HLA-A2 transgenic mice. Researchers demonstrated, taken as a whole, that CLIP binds to a wide variety of HLA-I molecules with surprising promiscuity. The discovery of CLIP’s involvement in the HLA-I antigen presentation pathway raises the possibility of a new processing route or immune escape mechanism, or it may indicate an abnormal process in leukemic cells (144).

The antigen delivery processing pathway of HLA-I and HLA-II complexes involves specific proteins. For HLA-II to deliver antigens, the invariant chain (II) peptide CLIP must stabilize HLA-II molecules by transiently and non-specifically adhering to different grooves on HLA-II. Scientists demonstrated an alternate mechanism for CLIP binding to HLA-I molecules on the surface of leukemic cells. Scientists found that AML cells that did not express HLA-II displayed the CLIP peptide on their plasma membrane. It was postulated that CLIP is directly involved in the HLA-I antigen presentation pathway due to the reduced HLA-I cell surface display in AML cells after Ii silencing. The HLA-I-specific peptide eluates from B-LCLs included five peptides derived from Ii, including two within the CLIP region. Experiments involving the binding of eluted CLIP peptide RMATPLLMQALPM to four distinct HLA-I supertypes (-A2, -B7, -A3, -B40) revealed an astonishing result. Shorter CLIP peptides demonstrated binding to all four supertypes, unlike in silico methods that solely anticipated binding to HLA-A2 or -B7. The administration of these peptides to HLA-A2 transgenic mice did not activate CTL responses. The results indicate that CLIP can bind to a diverse range of HLA-I molecules, which is noteworthy. The involvement of CLIP in the HLA-I antigen presentation pathway suggests a potential malfunction in leukemic cells. Still, it may also provide light on new processing routes or immune escape strategies (143, 145).

HLA-II antigen presentation is impacted by the regulation of normal and malignant cell peptide repertoires by the peptide editor HLA-DM and its antagonist HLA-DO. Balancing HLA-DM and HLA-DO means alloreactive T-cells may present leukemia-associated antigens and peptides differently, impacting graft versus host disease and anti-leukemia immunity. Utilizing a substantial quantity of bulk and single-cell RNA sequencing data from many archives, researchers examined the distribution and abundance of HLA-DM and HLA-DO in various human cell types and organs. Researchers attempting to analyze the dual function of HLA-II peptide editing in allogeneic hematopoietic cell transplantation (alloHCT) and their possible influence on its clinical success will benefit from the expression atlas produced (146).

The HLA-I and HLA-II complexes carry antigens through their processing routes using different proteins. By transiently and non-specifically attaching to different HLA-II peptide grooves, the peptide CLIP from the invariant chain (II) stabilizes HLA-II molecules before antigen loading, enabling antigen presentation. CLIP binds differently to HLA-I molecules on leukemic cells, according to researchers. In AML cells lacking HLA-II, the CLIP peptide was found on plasma membranes. The decrease in HLA-I cell surface expression in AML cells after Ii silencing shows that CLIP directly affects the HLA-I antigen presentation pathway. Five peptides from Ii and two from the CLIP area were found in B-LCL HLA-I peptide eluates. Four HLA-I supertypes (-A2, -B7, -A3, -B40) bound well to the eluted CLIP peptide RMATPLLMQALPM *in vitro.* Even while in silico approaches suggested exclusive binding to HLA-A2 or -B7, shorter CLIP peptides bound to all four supertypes. HLA-A2 transgenic mice were immunized with these peptides but did not produce CTLs. The findings showed CLIP’s versatility in interacting with HLA-I molecules. That CLIP is implicated in the HLA-I antigen presentation pathway implies a disturbance in leukemic cell processes. It may also reveal novel processing channels or immune escape methods (147).

A small number of malignant Hodgkin Reed-Sternberg (HRS) cells are present in primary classical Hodgkin lymphomas (cHL), which are characterized by a diverse infiltrate of immune and inflammatory cells (148). Several methods, primarily resulting from certain genetic defects, allow HRS cells to evade antitumor immunity. These include altered antigen presentation and enhanced PD-1 signaling. Researchers identified β_2_M mutations in both primary and HRS cell lines that hinder the production of the β_2_M/MHC-I dual protein complex on the cell surface (149). The expression of PD-1 ligands in HRS cells is influenced by the copy number variations of 9p24.1/CD274(PD-L1)/PDCD1LG2(PD-L2). A more advanced clinical stage and poorer progression-free survival (PFS) are associated with PD-L1/PD-L2 amplification after first-line (induction) therapy. Few details are known about the relationships between PD-L1/PD-L2 amplification, clinical outcome in cHL, and the enhanced expression of β_2_M, MHC-I, and MHC-II by HRS cells (150). The variables were examined in diagnostic biopsy specimens from 108 patients with cHL who were treated uniformly and followed up for an extended period. Researchers demonstrated that out of these patients, 79% (85/108) had reduced or nonexistent expression of β_2_m/MHC-I, and 67% (72/108) had reduced or nonexistent expression of MHC-II. Subjects without PD-L1/PD-L2 amplification or advanced stage showed a lower PFS if their β_2_m/MHC-I was diminished or nonexistent. There was no correlation between the result and reduced or nonexistent MHC-II. These findings provide further evidence that the biological response to conventional chemo and radiation treatment relies on MHC-I-mediated antigen presentation by HRS cells. Speculation about other ways of PD-1 blockage working in cHL is also prompted by the lack of β_2_m/MHC-I expression on HRS cells (151).

## 4 Peptide loading complex in breast cancer

As the cause of almost 41,000 deaths and 30% of all new cancer diagnoses in the US, BC continues to pose a danger to women’s health and well-being (152). The mortality rate from BC has decreased by 38% due to improvements in early identification and treatment, yet almost all patients who get metastatic illness will still die from it. These alarming numbers highlight the urgent need for new strategies in BC treatment to lessen the likelihood of recurrence and mortality (153). Evidence has been mounting regarding the immune system’s essential involvement in predicting BC patients’ responses to conventional treatment and their chances of long-term survival in recent years (154).

The sample for meta-analysis research included 3590 patients from eight different studies. Research on conventional HLA-Ia molecules (HLA-ABC) was the only one taken into consideration. Improved disease-free survival (DFS) was correlated with elevated HLA-I protein expression (HR 0.58, 95% CI 0.35-0.95, P = 0.03), especially in patients with triple-negative breast cancer (TNBC) (HR 0.31, 95% CI 0.18-0.52, P < 0.001). However, researchers revealed that there was no correlation with OS. Improvements in DFS, positivity for human epidermal growth factor receptor 2 (HER2), TNBC, high Ki-67 indices, and nuclear grades were all found to be associated with increased expression of HLA-I proteins, according to this meta-analysis. One possible predictive marker for BC is the immunological target HLA-I (155).

NLRC5 plays a pivotal role in cancer immunosurveillance by transactivating MHC-I, which is typically HLA-ABC in humans. However, the expressional and functional abnormalities of NLRC5 are a significant mechanism of immune evasion in many malignancies. In cancer immunotherapy and the fight against cancer immune evasion, NLRC5 promotion in conjunction with MHC-I augmentation is essential (156, 157). Scientists showed that the groups given 50 U/ml and 100 U/ml of IFN-γ had relative levels of NLRC5 mRNA, β_2_m mRNA, and HLA-ABC α HC mRNA that were dose-dependently higher than the control group. Protein levels of NLRC5 and β_2_m were noticeably more excellent in the groups treated with 50 U/ml and 100 U/ml IFN-γ, as well as HLA-ABC (positive rates) at different doses of IFN-γ, compared to the control group. Additionally, there was a dose-dependent tendency for both NLRC5 and HLA-ABC. This might be useful in cancer immunotherapy since it prevents cancer cells from escaping immunosurveillance, as NLRC5 is boosted in SKBR3 BC cells by IFN-γ along with overexpression in MHC-I (HLA-ABC) expression (158).

The protooncogene ERBB2 codes for a 185-kDa transmembrane glycoprotein that belongs to the epidermal growth factor receptor (*EGFR*) family of tyrosine kinases; it is more often known as HER2 or Neu (159). Since 20-30% of BCs have amplified and overexpressed HER2, it is a potential target for immunotherapeutic treatments using T-cell-based methods. Additionally, PD98059, an inhibitor of mitogen-associated protein kinases, elevated MHC-I expression on BC cell lines in a dosage-dependent way. BC cells may express more MHC-I if prescribed a medicine that inhibits the MAPK signaling pathway. A RAS/MAPK pathway-involved mechanism causes MHC-I downregulation in response to HER2-overexpression. Researchers provided further evidence that inhibitors of the RAS/MAPK pathway may enhance the production of MHC-I in BC cells (160, 161).

## 5 Peptide loading complex in lung cancer

LC is one of the most common cancers in the globe. Based on its shape and histology, LC may be classified into two main categories (1, 162). A little over 15% of newly diagnosed LC cases are small cell lung cancer (SCLC), but the vast majority (about 85%) are NSCLC. SCLC may be either extensive-stage (ES-SCLC) or limited-stage (LS-SCLC). Patients with LS-SCLC, which accounts for about 30% of SCLC cases, often undergo radiation therapy and chemotherapy based on platinum. The standard first treatment for ES-SCLC is chemotherapy alone, which consists of four to six cycles of carboplatin or cisplatin with etoposide. Approximately 70% of all SCLCs are ES-SCLC (163). The majority of NSCLCs were adenocarcinomas, squamous cell carcinomas, and large cell carcinomas (3, 164, 165). LC is caused by several factors, including smoking, radon gas, air pollution, asbestos, and heredity (166). LC is diagnosed using tumor markers such as cytokeratin 19 (Cyfra 21-1), neuron-specific enolase (NSE), and carcinoembryonic antigen (CEA). However, tumor markers are not appropriate for early diagnosis (167).

NSCLC with ALK translocations is currently treated with targeted therapy because of the molecular understanding of LC, which includes mutations in the *EGFR* TK domain and crizotinib (168). Given the known link between increased tumor- and stromal-infiltrating CTL numbers and better disease-specific survival in LC patients, immunotherapy that stimulates a CTL response against the cancer, such as peptide vaccines, may be a promising treatment approach. Researchers looked at HLA-I and II-presented peptides and gene expression patterns in surgically excised LC tissues to find (new) tumor antigens. Researchers also looked at the possibility that healthy donors can develop a CTL response to HLA ligands derived from these antigens. Researchers identified several potential candidates for tailored CTL recognition among the 170 HLA ligands they have examined. These targets might generate peptides from cyclin D1 or protein kinase, DNA-activated polypeptides, catalytic polypeptides, and lysed tumor cells loaded with peptides into CD8+ T-cells. This is the first *ex vivo* molecular characterization of HLA-I and II ligands taken from human LC tissues using the efficient identification of both known and new tumor antigens that may induce a CTL response (169).

A crucial gene in antigen-presenting cells, human leukocyte antigen-DP alpha 1 (HLA-DPA1), is involved in immunological modulation. Researchers set out to thoroughly examine the functions of HLA-DPA1 and how it relates to lung adenocarcinoma (LUAD). The effects of HLA-DPA1 expression on LUAD development and immunity were investigated by Ke Shi et al. (170) by a meta-analysis and bioinformatics. To further confirm HLA-DPA1’s roles in LUAD, researchers used the CCK-8, wound healing, and Transwell assays. Overexpression of HLA-DPA1 reduces the proliferation and development of cancer cells, while decreased expression is linked to a poor prognosis and immune infiltration in LUAD. Thus, HLA-DPA1 may be helpful as a treatment target for LUAD and a biomarker for prognosis (170).

It is increasingly evident that most malignancies exhibit deficiencies in the MHC-I antigen processing pathway and low levels of TAP and Tsn. Therefore, solutions to address these deficiencies are needed in immunotherapy efforts that target such tumors using CD8+ CTL. Researchers have discovered an antigen processing route that allows hydrophobic peptides produced cytosolically to be delivered without TAP. Researchers have demonstrated that numerous hydrophobic TAP-independent peptides can also be delivered in a Tsn-independent manner, as shown by the Tsn-negative cell line 721.220. However, the TAP-, Tsn-independent peptides were not seen when these tests were expanded to tumor cell lines generated from SCLC, which researchers demonstrated to be Tsn deficient in addition to TAP-negative. IFN-γ pretreatment of SCLC cells might address this lack of presentation. Alternatively, SCLC cells effectively presented the TAP-, Tsn-independent peptides by guiding them into the ER via an ER signal sequence. These findings led researchers to conclude that the TAP-independent mechanism for presenting hydrophobic peptides produces a low peptide concentration in the ER. This antigenic peptide concentration is insufficient for tumor cells lacking Tsn to bind to MHC-I molecules. Consequently, when creating immunotherapeutic strategies to target SCLC and other malignancies with anomalies in the MHC-I antigen processing pathway, it will be imperative to consider strategies that treat Tsn-defects (171).

Most tumors progressively show signs of abnormalities in the MHC-I antigen processing pathway, including low levels of TAP and Tsn. Therefore, methods to address these flaws are needed in immunotherapy efforts that use CD8+ CTL to target such tumors. Hydrophobic peptides produced from cytosol might be delivered without TAP thanks to a previously discovered antigen processing mechanism. Researchers showed that some of these hydrophobic TAP-independent peptides may also be given in a Tsn-independent fashion using the Tsn-negative cell line 721.220. Nevertheless, the TAP-, Tsn-independent peptides were not provided when these tests were expanded to tumor cell lines generated from SCLC, which researchers demonstrated to be Tsn deficient in addition to TAP-negative. Pre-treating SCLC cells with IFN-γ might address this lack of presentation. Or, the TAP-, Tsn-independent peptides were effectively delivered by SCLC cells using an ER signal sequence that guided them into the ER. Researchers deduced from these findings that the ER produces a low quantity of peptide in the TAP-independent route for the presentation of hydrophobic peptides and that this concentration of antigenic peptide is inadequate for tumor cells that also lack Tsn to load onto MHC-I molecules. Therefore, techniques that treat Tsn-abnormalities will be crucial to take into account for immunotherapeutic efforts to target SCLC and other tumors with defects in the MHC-I antigen processing pathway (129).

The highly aggressive neuroendocrine tumor SCLC exhibits moderate benefits with immune checkpoint blocking (ICB) and early-developed treatment resistance. A significant factor contributing to resistance against T-cell-based immunotherapies is the suppression of MHC-I expression. Using human SCLC cell lines and immunocompetent animal models, researchers assessed Lysine-Specific Demethylase 1 (LSD1)’s impact on MHC-I expression, functional antigen presentation, and immune activation in SCLC *in vitro* and *in vivo.* By explicitly blocking LSD1, researchers were able to promote the transcription of genes related to the antigen presentation pathway and reestablish MHC-I cell surface expression in SCLC. Blocking LSD1 makes tumor cells more intrinsically immunogenic, increases interferon signaling, and makes SCLC cells more susceptible to cytolysis by T lymphocytes that are limited to MHC-I. In refractory SCLC models, combining ICB with an LSD1 inhibitor enhances the anti-tumor immune response. The results collectively indicate that LSD1 serves as a significant regulator of MHC-I antigen presentation, supporting the hypothesis that the combination of LSD1 inhibitors and immune checkpoint blockade could enhance therapeutic responses in patients with SCLC. A possible explanation for SCLC’s poor response to ICB might be the epigenetic suppression of MHC-I. Recently, researchers showed that LSD1 regulates MHC-I antigen presentation in SCLC, a largely unknown activity. In SCLC, blocking LSD1 allows for MHC-I-restricted T-cell cytolysis, immunological activation, and an improved anti-tumor immune response to ICB (172).

## 6 Peptide loading complex in other cancer

Regarding cancer diagnoses worldwide, CRC is the third most common diagnosis, with stomach cancer taking the fifth position. As a result of advancements in early detection techniques and longer lifespans, synchronous and metachronous cancers—whose estimated incidences vary from 2.5% to 3.4%—have become more common. Interestingly, CRC is the most common synchronous and metachronous event in individuals with gastric cancer, with lung and liver cancers coming in second and third, respectively (173).

W Schmidt et al. have devised a method to produce a highly effective immunogenic vaccine: This method, which scientists call “trans-loading,” entails combining MHC-I-positive cancer cells with foreign, nonself peptide ligands. Cancer CT-26 (H2-Kd), melanoma M-3 (H2-Db haplotype), and H2-Kd were given as irradiation vaccines, together with influenza virus peptides that were MHC-matched. Mice treated with this method successfully eradicated a tumor containing live M-3 melanoma cells. The CT-26 colon cancer and the B16-F10 melanoma showed high efficacies against tumor challenge, suggesting that this new vaccination might have broad applicability. This idea might be used to treat human tumors using foreign peptide ligands tailored to a particular MHC-I haplotype (130, 174).

Giving a patient with gastric cancer a composition including activated T-cells—a population of lymphocytes that can identify and specifically target patient cells that produce a peptide aberrantly—is one way to treat the patient. Activated T-cells that can identify patient cells that aberrantly express a peptide are included in a pharmaceutical composition. The composition is intended to treat gastric cancer patients by having the T-cells bind to the peptide in a complex with an MHC-I molecule and a pharmaceutically acceptable carrier. One way to treat a patient with stomach cancer is to give them a composition that has a peptide in a salt that their doctor approves. This will trigger a T-cell response to the gastric cancer (175).

Using a novel monoclonal anti-pan HLA-I HC antibody (EMR 8-5), which interacted with paraffin-fixed sections, researchers examined the predictive significance of HLA-I molecules in patients undergoing radical cystectomy for muscle-invasive bladder cancer. Researchers demonstrated that subjects from 65 patients who had radical cystectomy, pelvic lymph node dissection, and clinically confirmed muscle-invasive bladder cancer were immunohistochemically stained for HLA-I molecules using the mAb EMR 8-5. Neoadjuvant treatment was not administered to these patients. Researchers examined the clinicopathological and predictive importance of HLA-I expression. According to immunohistochemical examination, 22 invasive bladder tumors had HLA-I down-regulation. This downregulation and clinicopathological parameters, including grade, nodal status, or pathologic stage, were not correlated, and compared to patients whose tumors were downregulated, those whose tumors were HLA-I positive had a much better recurrence-free survival rate. After a cystectomy, individuals with bladder cancer who had HLA-I expression had a substantial impact on their chance of not having a recurrence, according to multivariate analysis. Researchers showed that HLA-I was down-regulated in tumor cells in around a third of the patients. People who develop muscle-invasive bladder cancer after a cystectomy may be able to use HLA-I expression as a prediction (176).

A meta-analysis was conducted by Hadis Najafimehr et al. to (177) ascertain the function of classical HLA-I in patient survival prediction. Furthermore, researchers assessed the potential association between HLA-I and several clinicopathological variables. Researchers identified any associations between HLA-I and OS or RFS in gastrointestinal cancer patients by reviewing published research that examined the influence of HLA-I expression on these malignancies. Effect sizes with a 95% confidence interval were hazard ratios (HR) and odds ratios (OR). The analysis included 10 trials with a total of 1,307 patients. Overexpression of HLA-I was favorably associated with OS (HR:0.72; 95% CI:0.53-0.96) and showed little connection with RFS (HR:0.70; 95% CI:0.46-0.08), according to the combined findings. Overexpression of HLA-I is negatively linked to lower tumor differentiation [OR: 0.53; 95% CI (0.43-0.81)] and advanced cancer stages [OR: 0.29; 95% CI (0.13-0.64)]. There was a positive correlation between HLA-I overexpression and OS prognosis, although there was likely little effect on RFS (177) (**Table 1**).

The prognostic significance of human leukocyte antigen-G (HLA-G) is still up for debate despite reports of abnormal expression in CRC. Accordingly, researchers used the literature and the datasets from The Cancer Genome Atlas (TCGA) to conduct a meta-analysis that will evaluate the predictive usefulness of HLA-G in patients with CRC. Researchers meta-analysis found a strong correlation between LA-G expression and poor OS in CRC. Nevertheless, when comparing researchers’ findings to the TCGA data in CRC, researchers discovered that the prognostic significance did not align. That is why researchers still need further studies to prove that HLA-G has a predictive function in CRC (178).

In order to determine if there is a correlation between HLA-G expression and solid tumor outcomes, Jorge Bartolome et al. conducted a meta-analysis and systematic review. The inclusion criteria were satisfied by 25 research. Data were collected from 4871 patients who reported OS and 961 patients who reported DFS. There was a greater risk of gastric, pancreatic, and CRCs being related to HLA-G expression, which was associated with poorer OS (HR 2.09, 95% CI = 1.67 to 2.63; P <.001). The most used antibody (4H84) and other detection techniques showed no statistically significant changes, according to the researchers. An association between HLA-G expression and DFS was observed. However, it did not reach statistical significance (179).

## 7 Compare the PLC-based approaches with CAR-T cell therapy in cancer

Recently, chimeric antigen receptor (CAR) T-cell therapy—which the U.S. Food and Drug Administration has authorized—has shown promise in treating mantle cell lymphoma (MCL), DLBCL, and r/r B-ALL. Research on the B-cell maturation antigen (BCMA) in multiple myeloma has shown promising results, with reversible toxic consequences such as pancytopenia and cytokine release syndrome (CRS). Nevertheless, there has been uneven toxicity and effectiveness (180). CAR-T cells must initially travel to tumor locations in order to attach to their target proteins on the tumor surface. This is a basic need for the effective use of T-cell immunotherapy. In contrast to hematological malignancies, the immunosuppressive microenvironment often significantly restricts T-cell trafficking to and infiltration into tumor sites in solid tumors. Additionally, some chemokines released by solid tumors, such as CXCL1, CXCL12, and CXCL5, inhibit T-cells from migrating to and penetrating the tumor lesions, in contrast to hematological malignancies that are simple for CAR-T cells to target and reach. CAR-T cells’ intended immuno-cytotoxicity to kill tumor cells is severely hampered by their inability to traffic and infiltrate into tumor locations due to the absence of matching chemokine receptors present in T-cells. T-cells must thus be altered to express a chemokine receptor that corresponds to the relevant tumor-derived chemokine in order to get beyond this barrier (181).

An overactive immune system and systemic inflammatory response are the results of the vast quantities of cytokines released when CAR-T cells destroy tumor cells. Fever, chills, and headache are signs of mild CRS; hypotension, respiratory distress, and organ insufficiency are indications of severe CRS, which may be fatal. Further complications include organ function degradation due to CAR-T cell persistent cell growth and anemia, thrombocytopenia, and leukopenia caused by CRS, which may increase the risk of infection and spontaneous bleeding. Reduced numbers of lymphocytes and other immune cells may result from cytokine release. The total number of immune cells, including T-cells, B cells, and NK cells, decreases due to lymphodepletion, resulting in cytopenia (182–184).

One way that CAR T-cells avoid some of the problems with the TCR-based method is by targeting cells in a way that is not reliant on MHC and DCs. A CAR differs significantly from a TCR in its ability to attach to a target and activate T-cells. Here are a few examples: the amount of MHC molecules, the existence of co-receptors, the TCR avidity for a particular pMHC, and the moderate TCR affinity for the associated MHC peptide complex, as opposed to the high antibody affinity in a CAR. The TCR’s variability is restricted since it can only identify peptide antigens within the setting of a certain MHC. One advantage of CAR T-cells based on antibodies is their ability to target antigens with varying shapes and compositions, including peptides, carbohydrates, and inorganic substances. Furthermore, TCRs have an innate cross-reactivity with endogenous antigens. As of right now, there are considerably more possible CAR targets than their MHC-presented counterparts that TCR-modified T-cells can identify. Researchers examined the benefits and drawbacks of CAR T-cells’ ability to recognize targets without MHC and highlighted the most critical advancements in cancer treatment early-stage clinical studies (185).

Problems with poor antigen density, tonic signaling, and antigen loss are obstacles to CAR-T cell treatment. Traditional CAR T-cell treatment cannot reach most tumor-specific antigens because they are either secreted or located inside cells. Researchers utilized alpha-fetoprotein (AFP), a specific marker for liver cancer, to test the hypothesis that peptide-MHC complexes could be targets for CAR T-cell therapy against solid tumors. This is because all intracellular and secreted proteins are processed into peptides and presented by MHC-I on the surface of tumor cells (184, 186, 187).

Researchers created ET1402L1-CAR (AFP-CAR), an entirely human chimeric antigen receptor, which has exceptional selectivity and specificity for the AFP158–166 peptides complexed with HLA-A*02:01. T-cells expressing AFP-CAR spared cells from various tissue types that were negative for either expressed protein. Still, they specifically degranulated, produced cytokines, and lysed liver cancer cells that were HLA-A*02:01+/AFP+. In SCID-Beige mice (n = 8 per group), intratumoral injection of AFP-CAR T-cells markedly reduced both Hep G2 and AFP158-expressing SK-HEP-1 tumors. Furthermore, Hep G2 tumor-bearing NSG mice that receive intravenous AFP-CAR T-cell injection have a significant and quick suppression of tumor development (n = 6). Lastly, AFP-CAR T-cells demonstrated strong antitumor efficacy in a well-established intraperitoneal liver cancer xenograft paradigm (n = 6). Researchers showed that a potent antitumor response may be produced by CAR T-cell immunotherapy that targets intracellular/secreted solid tumor antigens. Researchers’ method provides a potential new path for liver cancer immunotherapy and broadens the range of antigens accessible for redirected T-cell treatment against solid tumors (187).

Although mHAg-specific TCRs have been created in genetically engineered T-cells, their functionality may be jeopardized if endogenous and exogenous TCR chains generate mismatched chimeric TCRs. A different strategy is the creation of chimeric antigen receptor (CAR) T-cells with TCR-like specificity, whose intracellular and transmembrane domains send their activation signals instead of competing with endogenous TCRs for CD3 complexes. However, it has been shown that high-affinity CAR-T cells have poor sensitivity and specificity when it comes to recognizing low-density antigens (188, 189). The groove of an HLA molecule is where a minor histocompatibility antigen (mHAg) may be found, which is made up of different genes from the donor and the recipient. The graft-vs.-leukemia effect, which occurs after donor lymphocyte infusion, has demonstrated the therapeutic potential of T-cells specific to mHAgs against recurring hematologic malignancies. Still, this effect only applies to recipient-target hematopoietic cells, such as leukemia cells (190). According to current research, the most crucial goal in adoptive cell therapy is the rapid development of TCR-like CAR-T cells that use an all-in-one chimeric receptor with adjustable intracellular signaling domains. Chimeric antigen receptor-T cells outperform modified TCR gene-introduced T-cells in situations where costimulatory signals from target cells are either not present or are downregulated. This is due to their built-in signaling domain. This alternative is safer and more promising than CAR-T therapies; nevertheless, due to a number of advancements in TCR gene-introduced T-cells (190, 191).

The development of efficient screening systems, including a range of panel peptides, HLA-typed cell lines, and animal models to assess the efficacy and toxicity of TCR-like antibodies, along with meticulous preclinical experiment planning, is essential for the acquisition of TCR-like antibodies that could have therapeutic uses. In contrast to gene-modified T-cells and other passive immunotherapies, current studies are evaluating active immunotherapies such as DC-based peptide or DNA vaccines with or without adjuvants. Since gene-modified cells are subject to stringent controls, these methods are believed to be more practical and cheaper. Nevertheless, due to the lack of publicly available phase I/II clinical data, including a phase I dosage assessment trial for a HA-1 mHAg vaccination, it is premature to compare the two main methods. To determine the best approaches and patient groups for mHAg-targeted immunotherapy, more intervention-specific studies are required (189, 191–193) (**Table 2**).

**Table 2:** Compare the PLC-based approaches with other emerging cancer therapies, including CAR-T cell therapy.

Comparative cases	PLC-based therapy	CAR-T cell therapy	Ref
**Targeting mechanism**	Tumor cells may evade immune monitoring when mutations in MHC-I molecules alter antigen presentation. In addition, MHC-I displays antigens to T-cells. To prevent tumor progression, the immune system may identify tumor-specific neoantigens and eradicate them thanks to HLA-I molecules, which deliver endogenous antigenic peptides to cell surfaces.	By facilitating the binding of target cell surface antigens via a single-chain variable fragment (scFv) recognition domain, CAR T-cells accomplish MHC-unrestricted tumor cell killing. For CAR T-cells to carry out their effector role, they must first create a non-classical immunological synapse (IS) upon contact. Antigens are proteins found on the surface of cancer cells that the CARs identify and attach to.	(126, 194, 195)
**Antigen specificity**	Intracellular and neoantigens are part of the broad category. HLA typing It is common for MHC-I to deliver neoantigens to CD8+ T lymphocytes in a cell-specific manner.	Applicable only to antigens found outside of cells (such as CD19 and BCMA). At now, CAR-T cells are being directed toward antigens that are unique to a particular lineage, such as CD19 or B cell maturation antigen (BCMA). Unfortunately, normal cells expressing these antigens may be lethally targeted, making lineage-specific antigens useless as targets in the majority of malignancies.	(196, 197)
**Immune evasion**	Researchers focused on malignancies and the downregulation of MHC. Loss of MHC I antigen presentation machinery (APM) is one way malignancies might avoid immune regulation, as MHC-I molecules are not necessary for cell survival.	Antigen escape, such as the loss of a CAR target, may reduce this. Antigen escape is one typical obstacle that drastically lowers the efficacy of CAR-T cell treatments.	(88, 198)
**Efficacy in solid tumors**	It has promise, although it is still in the development phase. Furthermore, these treatments reduce off-target immune responses against healthy tissues by zeroing in on tumor-specific or neoantigens, as shown by MHC-I.	Solid tumors have a low success rate. Most CARs do not discriminate between tumor and non-tumor cells when recognizing them; CAR targets are TAAs, which are overexpressed on tumor cells relative to normal cells.	(199, 200)
**Side effects**	Cytokine release syndrome (CRS) is rarer. Regrettably, reduced MHC-I expression in tumors is related to worse treatment efficacy and therapeutic resistance development across a range of cancer types.	Potentially harmful effects on the nervous system and central nervous system injuries. Chronic autoimmune response (CAR-T) cells cause organ dysfunction due to their uncontrollable cell proliferation. In addition to increasing the risk of infection and inducing spontaneous bleeding, CRS may cause anemia, thrombocytopenia, and leukopenia.	(182–184, 201, 202)
**Duration of effect**	It may provide immune system monitoring over an extended period.	Reinfusion may be necessary, or the persistence may be low.	(201, 203)
**Manufacturing complexity**	Simpler; uses the body’s immune system to its advantage.	Complex; calls for engineering and isolation of T-cells.	(202)
**Important advantages**	The presentation of intracellular antigens by MHC-I molecules makes MHC-I-based treatments applicable to a broad variety of tumor forms. A variety of HLA molecules is used in HLA treatments, which may allow for personalized methods according to each patient’s genetic makeup. By reducing MHC-I expression, tumor cells are able to elude detection by the immune system. These treatments reduce harmful immune responses to healthy tissues by zeroing in on tumor-specific or neoantigens shown by MHC-I.	Leukemia and lymphoma have been tremendously helped by CAR-T cells. Rapid antigen targeting is possible with CAR-T.	(204, 205)

## 8 Clinical studies related to PLC-based approaches in the treatment of cancers

The ability of DC loaded with wild-type vs modified gp100 peptides with more remarkable binding affinities to elicit an immunological and clinical response in patients with advanced melanoma was examined in this research by Lesterhuis WJ et al. In patients with metastatic HLA-A2.1(+) melanoma, mature DC loaded with keyhole limpet hemocyanin (KLH), tyrosinase peptide, and either wild-type (15 patients) or modified (12 patients) gp100 peptides were administered intravenously (on average 25 × 10(6) DC) and intradermally (on average 11 × 10(6) DC). Every patient who received the vaccination had a strong humoral or proliferative T-cell response against KLH. Tetramer and functional analysis were used to track Gp100-specific T-cell responses in post-treatment delayed-type hypersensitivity (DTH) skin biopsies. Two out of fifteen patients who received the wild-type gp100-loaded DC vaccine had antigen-specific T-cells, compared to one out of twelve who received the modified peptide-loaded DC vaccine. With two patients in each group exhibiting long-term (>8 years) complete responses, these three patients also showed the most significant clinical response. Researchers find that a small percentage of patients with metastatic melanoma may have long-term clinical responses after receiving peptide-loaded DC immunization and that using modified gp100 peptides for DC loading instead of wild-type peptides does not provide a meaningfully stronger immune response (206).

In patients with metastatic melanoma, the DCs vaccination identified hitherto unidentified HLA-I-restricted neoantigens and increased naturally existing neoantigen-specific immunity. Mass spectrometry verified the presence of neoantigens by HLA-A*02:01 in human melanoma. In terms of TCR-β use and clonal composition, vaccination facilitated a varied repertoire of neoantigen-specific TCRs. Researchers’ findings showed that antitumor immunity’s clonal diversity and antigenic breadth are enhanced by vaccination targeting tumor-encoded amino acid changes (ClinicalTrials.gov NCT00683670) (207).

In order to link the findings with transcriptional and genomic studies as well as the clinical response to anti-CTLA-4, anti-PD-1, or combination therapy, researchers looked at the expression of MHC-I and II proteins on tumor cells from melanoma patients who had not received treatment before. Primary resistance to anti-CTLA-4, but not anti-PD-1, treatment was predicted by the majority (>50% of cells) or total loss of melanoma MHC-I membrane expression, which was seen in 78 out of 181 cases (43%). It was linked to transcriptional suppression of HLA-A, HLA-B, HLA-C, and *β2M*. 55 out of 181 patients (30%) had melanoma MHC-II membrane expression on >1% cells, which was linked to IFN-γ and IFN-γ-mediated gene signatures and indicated a response to anti-PD-1 treatment but not anti-CTLA-4 therapy. Researchers inferred that strong melanoma MHC-I expression is necessary for the primary reaction to anti-CTLA-4. Contrarily, in cases where MHC-I is compromised, the primary response to anti-PD-1 is associated with pre-existing immune activation via IFN-γ, which includes tumor-specific MHC-II expression and components of innate immunity. The fact that antitumor immunity has various requirements for melanoma-specific antigen presentation might be one reason why combination checkpoint blockage is more effective (208).

One of the most challenging side effects of unrelated donor HCT is graft-versus-host disease (GVHD) (209). The highly polymorphic MHC class I chain-related protein A (MICA), encodes a stress-induced glycoprotein that is mainly generated on epithelia. The cytotoxic lymphocyte-expressed invariant activating receptor NKG2D interacts with MICA, which is located in the MHC next to HLA-B. Thus, MICA has all the essential qualities of a valid antigen for transplantation. Using high-resolution sequence-based MICA genotyping, researchers retrospectively studied the clinical effect of MICA mismatches in a multicenter cohort of 922 unrelated donors of HLA-A, HLA-B, HLA-C, HLA-DRB1, and HLA-DQB1 alleles in 10/10 HCT couples. There were 113 mismatched pairs (12.3%) out of 922 in MICA. An increased risk of nonrelapse mortality and acute GVHD grade III–IV were significantly associated with MICA mismatches. A potential graft-versus-leukemia impact was suggested by the fact that the decreased risk of relapse matched the elevated risk for GVHD. In conclusion, choosing a donor who is MICA-matched wherever feasible has a major impact on significant clinical outcomes of HCT when a considerable decrease in GVHD is crucial. Because of the close linkage disequilibrium between MICA and HLA-B, finding a donor who matches MICA is easily achievable in clinical settings (210).

Immune checkpoint blockers (ICBs) cause either fast progression (primary resistance) or long-lasting benefits (secondary resistance) in patients with advanced NSCLC. Following ICB failure, the cancer vaccine OSE2101 may boost antitumor-specific immune responses. Assessing ATALANTE-1’s safety and effectiveness in these patients was its primary goal. The purpose of the two-step open-label ATALANTE-1 research was to compare the safety and efficacy of OSE2101 to standard-of-care (SoC) chemotherapy (CT). Individuals with advanced NSCLC who tested positive for the HLA-A2 and had no discernible changes, failing either sequential or concurrent CT and ICB, were randomized 2:1 to either OSE2101 or SoC (docetaxel or pemetrexed). OS was the primary outcome. An interim OS futility analysis was scheduled in line with Fleming’s design. The decision to immediately stop the accumulation caused by the 2019 coronavirus infection (COVID-19) was decided at the interim review in April 2020. The final analysis included all patients as well as a subgroup with ICB secondary resistance, which was defined as failure after 12 weeks of second-line ICB monotherapy without improvement. When compared to CT, OSE2101 increased survival. It decreased safety for patients with advanced NSCLC who tested positive for HLA-A2 and who had developed resistance to previous treatments. Additional investigation into this group of people is required (211).

Melanoma-associated Antigen A4 (MAGE-A4) is only expressed in immune-privileged tissues; it belongs to the MAGE protein family of cancer/testis antigens (212, 213). Patients with relapsed/refractory metastatic solid tumors expressing MAGE-A4, such as head and neck cancer, ovarian cancer, and synovial sarcoma (SS), participated in a multicenter, dose-escalation, phase 1 study by David S. Hong et al. (NCT03132922) (214). According to exploratory research, afami-cel infiltrates tumors and activates adaptive immune responses via an interferon-γ-driven mode of action. Furthermore, afami-cel has a favorable benefit-risk profile, exhibiting prompt and long-lasting effects, particularly in patients with metastatic SS. MAGE-A4-specific T-cells had a satisfactory safety profile in a phase 1 dose-escalation experiment, including patients with nine distinct solid tumor types. Patients with synovial sarcoma had a promising overall response rate, according to the researchers. The findings merit further testing in larger research, even if the limited trial size restricts the inferences that can be made (214).

Patients who test positive for HLA-A2 or -A24 are the primary focus of peptide-based cancer vaccines currently under development by researchers. A phase I clinical trial of peptide vaccines targeting six distinct HLA-A types in cancer patients was thus undertaken to circumvent this restriction. Patients have to test positive for the HLA-A2, -A24, or -A3 (A3, A11, A31, or A33) supertype and had previously failed traditional cancer treatments to be considered. Patients with HLA-A2(+), HLA-A24(+), and HLA-A3(+) alleles were given a different set of eight candidate peptides for a total of twenty-four peptides prior to vaccination. By taking into account the patients’ HLA types and pre-existing levels of IgGs to the candidate peptides, the vaccine peptides were personalized from the candidate pool. This research included seventeen patients. No patients had serious adverse effects as a result of the peptide vaccines, and they were well-tolerated overall. Eleven out of thirteen instances showed an increase in CTL responses. In contrast, ten out of thirteen cases showed an increase in IgG responses specific to the vaccine peptides. Due to its immunological reactions to the vaccinated peptides and their tolerability, this novel vaccine is suggested for phase II clinical trials (215).

Tumors expressing New York esophageal squamous cell carcinoma-1 (NY-ESO-1) are responsive to TCR-T cell treatment; however, a safe and effective TCR-T cell therapeutic regimen is still needed (212). Results from a phase 1 IND clinical trial, including TCR affinity-enhanced specific T-cell therapy (TAEST160001) for the treatment of NY-ESO-1, were published by Pan et al. After 3 days of dose-reduced lymphodepletion with cyclophosphamide (15 mg/kg/day) and fludarabine (20 mg/m2/day), patients who are enrolled in the study get an infusion of TAEST16001 cells (216). After the adoptive transfer, modest doses of IL-2 injection are used to maintain the TCR-T cells. Researchers demonstrated that there were no significant adverse effects associated with the medication in the 12 individuals who were given the regimen. A total of 41.7% of people have responded. The median response length is 13.1 months, while progression-free survival is 7.2 months on average. Pan et al. proved that TAEST16001 cells were safe, effective, and survived in patients with advanced soft tissue sarcoma who had the HLA-A∗02:01 gene mutation. According to ClinicalTrials.gov (NCT04318964), researchers demonstrated that TAEST16001 cell treatment for advanced soft tissue sarcoma expressing the NY-ESO-1 antigen is well-tolerated and has potential anti-tumor actions (216).

An essential component of fetal-maternal immunological tolerance is the non-classical MHC-I protein known as human leukocyte antigen-G (HLA-G) (217). As a first-of-its-kind immunoglobulin (Ig)G1 bispecific antibody, JNJ-78306358 was created by researchers to treat advanced solid cancers that express HLA-G. By binding to both the α3 domain of HLA-G isoforms on tumor cells and the CD3 receptor complex on T-cells at the same time, JNJ-78306358 facilitates the creation of immunological synapses, which allows cytotoxic T-cells to destroy tumor cells, and it also reduces the immunosuppressive tumor microenvironment (218, 219). This dose-escalation research assessed the pharmacokinetics, pharmacodynamics, and safety of JNJ-78306358 in patients with advanced solid tumors, as well as its preliminary anticancer efficacy. The study included adult patients whose solid tumors had spread or were not amenable to surgery and who also had a high frequency of HLA-G expression. Researchers showed that inducing cytokines and T-cell activation were pharmacodynamic effects of JNJ-78306358. The ability to raise the dosage of JNJ-78306358 to levels where it may be effective was hindered by CRS-related toxicities, such as pneumonitis and elevated transaminases (NCT04991740) (220).

## 9 Future directions

Peptides are increasingly being developed for use as therapeutics to treat many diseases, including cancer. The benefits of therapeutic peptides include reduced toxicity and target selectivity. A peptide’s ability to inhibit cancer may stem from its direct binding to its target or via its conjugation with a radionuclide or chemotherapeutic treatment to direct the agent’s actions specifically toward cancer cells. Peptides may penetrate the cell membrane directly or be directed to proteins on the cell surface, where peptide-protein interactions can start the internalization of the complex. Peptides can cause cell death by various methods, such as immunological modulation, disruption of cell signaling pathways, cell cycle control, DNA repair pathways, apoptosis, tumor angiogenesis inhibition, membrane breakdown, consequent necrosis, or cell death pathways. While there are numerous benefits to employing peptides as treatments, peptides have the drawback of being readily broken down by proteases after they are provided and sometimes having trouble getting absorbed into the bloodstream, depending on how they are supplied (221). The advances in modification chemistry and analytical technology over the last several decades have significantly advanced the creation of peptide medicines. The newly developed peptide pharmaceuticals have been enhanced through various biochemical techniques in therapeutic, diagnostic, and drug-delivery tactics. Scholars discovered that it supports overcoming the intrinsic constraints of peptides and advancing their applications further. The development of peptide-drug conjugates (PDCs) has laid the groundwork for the new era of tailored peptide pharmaceuticals by using target-oriented peptide moieties as a vehicle for lethal payloads by conjugation with cleavable chemical agents (222, 223).

The development of therapeutic CD8+ T-cell-based cancer vaccines and adoptive T-cell therapies is hindered by the lack of tumor-specific antigens (TSAs) that bind to MHC-I but do not evade T-cell self-tolerance. Most cancers have mutations, and the present approaches primarily focus on producing tolerance-breaking TSAs with a greater affinity for either MHC-I molecules or TCRs by predicting “neoantigens” in silico. These neoantigens are the product of alterations in annotated genes. The approach’s poor success ignited the search for TSAs derived from non-tolerized retroelements, genetic fusion events, or mRNA splice variants. Furthermore, T-cells collaborate with other immune cells to perform immunosurveillance. Lysing MHC-I-deficient cells, NK prevents MHC-I-mediated escape. Although they may not have as much direct effect, other immune cells such as B cells, DCs, macrophages, and CD4+ T-cells may have a significant pro-tumor and antitumor effect. Modulating several populations of innate and adaptive immune cells will be necessary to optimize immunotherapy. Comprehending and regulating therapeutic T-cell responses and immunoevasion need knowledge of immunity down to unique tumor-specific peptides. In cancer, changes to antigen presentation may influence some peptides but not MHC-I levels or the specific allomorph of MHC-I that presents the antigen. This means that the most extreme cases of immunoediting may be all that is now understood about immunoevasion. At the same time, many more nuanced alterations in the synthesis of tumor-specific peptides go undetected. The outcomes of these endeavors will have far-reaching implications for cancer immunotherapy. Still, they will also inform important questions about protein synthesis, degradation, trafficking, and all aspects of MHC-I immunosurveillance, including infections, autoimmune diseases, and tissue transplantation (224).

Important players in the tumor microenvironment include MHC-I and its associated molecules. MHC-I’s traditional antigen presentation roles preserve CD8+ T-cells’ fundamental anticancer immunity, which is vital in fighting against tumor cells. However, recent research has typically shown that MHC-I is cancer’s primary immunological escape mechanism. In the context of cancer, many elements cogently alter MHC-I expression and function. Pathways connected to MHC-I might be a promising target for cancer immunotherapy collaboration. Nonetheless, it is necessary to identify and clarify the key molecules that alter the MHC-I working orientations and their precise processes (126). Limitations mean that not all peptide/MHC complexes associated with a disease state will be found using indirect discovery techniques, and some unrelated peptide/MHC complexes may even be found. For example, an immune-centric approach to indirect discovery identified a peptide/HLA-A*02:01 complex unique to melanoma, which contains the human telomerase peptide hTERT_540–548_ (ILAKFLHWL). This result has not been replicated using other techniques. Vaccination or *in vitro* activation of CTLs resulted in CTL clones that specifically recognized hTERT540–548/HLA-A*02:01; nevertheless, results concerning the clones’ ability to lyse hTERT-expressing tumor cell lines are conflicting. Additionally, the hTERT_540–548_ peptide was not detected by mass spectrometry analysis of peptides that were directly isolated from the HLA-A*02:01 molecules of several hTERT-positive tumor cell lines, and proteasome processing experiments conducted *in vitro* did not result in the production of the hTERT_540–548_ peptide (unpublished data). The hTERT_540–548_/HLA-A2 ligand generated controversy among researchers, who emphasized the need to verify epitopes discovered indirectly (225).

One significant element assisting in tumor immune evasion is the downregulation of MHC-I. However, immune evasion of cancers is a complex process, as shown by the demonstrated resistance of solid tumors to CAR-therapy and by IFN-γ-mediated induction of PD-L. The authors thus postulate that in order to prevent tumor immune evasion, combined treatment that targets several tumor-immunomodulatory pathways at the same time would be required. Numerous immunomodulatory medication combinations, such as CPI in conjunction with STAT3 inhibitors or STING agonists, have encouraging outcomes in (pre-)clinical research (201, 226–228).

Tumors may avoid immune monitoring when they lose the ability to display MHC-I antigens, which is a typical occurrence in cancer. Even while structural genetic changes like β_2_m deletion cause MHC-I loss that cannot be undone, downregulation of MHC-I after transcription can be undone. Hence, for immunotherapies to work better, new ways to increase tumor-specific MHC-I (tsMHC-I) expression are needed (205). The IFN signaling pathway is one possible method by which tsMHC-I is upregulated. Numerous antigen presentation genes, including MHC-I and TAP, have been shown to express more when IFN-γ is present (205, 229). A transcriptional regulator of MHC-I and associated genes is NLRC5, a member of the NLR protein family (nucleotide-binding domain, leucine-rich repeat). Additionally, NLRC5 nuclear distribution and an intact nuclear localization signal are prerequisites for *MHC-I* gene activation via NLRC5-mediated mechanisms. Immediate effects on MHC-I expression and MHC-I-mediated antigen presentation are anticipated to result from adjustments to the cellular location of NLRC5. Therefore, NLRC5 could be an excellent target for transplant medicine-related modification of MHC-I antigen presentation (230). In SK-BR-3 BC cells, IFN-γ promotes NLRC5 with overexpression of HLA-ABC, which may help prevent cancer evasion from immunosurveillance and aid in cancer immunotherapy (158). The potentially fatal toxicities experienced by patients make the use of IFN in cancer treatment limited despite the fact that they may improve immunotherapy response. Through antibody-directed, dose-dependent cytokine release at the tumor-specific location, one potential way to use IFNs for therapeutic overexpression of tsMHC-I without the accompanying toxicity is to use this strategy. However, large-scale clinical studies have not yet explored this method (205).

The expression of MHC-I, β_2_M, and other APM components in cancer has been shown to be influenced by a number of oncogenic pathways, including the c-MYC, n-MYC, HER2, MAPK, and EGFR. It is hypothesized that stimulation of the MAPK pathway reduces IRF1 activity and STAT1 expression, which in turn affects MHC-I expression. It has been shown that the MEK inhibitors trametinib and cobimetinib boost STAT1 phosphorylation and IRF1 expression in human keratinocytes (201). Scientists examined the effects of EGFR tyrosine kinase inhibitors (TKIs) on MHC-I expression in NSCLC cell lines. Treatment with certain EGFR-TKIs increased MHC-I expression on both the mRNA and cell surface protein levels in cells that tested positive for EGFR mutations, including cells with the T790M secondary mutation. Since the phosphatidylinositol 3-kinase (PI3K) inhibitor buparlisib failed to increase MHC-I expression in response to EGFR activation, it seems that the down-regulation of MHC-I expression is mediated via the MEK-ERK pathway. The ERK kinase MEK inhibitor trametinib, however, was successful. Upon immunohistochemical analysis of EGFR-mutated NSCLC samples collected both before and after EGFR-TKI therapy, it was shown that MHC-I, the quantity of infiltrating CD8+ T-cells, phosphorylated EGFR, and ERK were all down-regulated, whilst PD-1 ligand one expression was up-regulated. Immunotherapy is ineffective against NSCLC tumors in part because the MEK-ERK pathway is blocked by EGFR mutational activation, which prevents MHC-I synthesis. Further research is required to fully understand the immune response to EGFR-mutated NSCLC and the signaling cascade including EGFR, MEK, and ERK (231).

According to Yi Bao et al. (232), the expression of ubiquitin-like modifier activating enzyme 1 (UBA1) had the most negative correlation with effector CD8+ T-cell-related characteristics. In ICB cohorts, poor survival and treatment resistance were highly predicted by high UBA1 expression. According to functional tests, UBA1 stimulated tumor development by mediating immune escape. Additionally, Uba1 overexpression or deletion significantly reduced or boosted functional intratumoral CD8+ T-cells, respectively, according to immune profile analysis. Crucially, in some syngeneic animals, TAK-243, a specific UBA1 inhibitor, greatly enhanced ICB’s synergy. Mechanistically, the UBA1-STUB1 axis’s reduction or inactivation increased important immune modulators such as CXCL9, CXCL10, and MHC-I, improved IFN-signaling and stabilized a crucial interferon pathway component (JAK1) (232).

Research into other methods of increasing tsMHC-I expression in an effort to reduce immunotherapy resistance is ongoing. Radiotherapy and chemotherapy, the gold standards of cancer treatment today, are recognized to stimulate immunogenic cell death without inducing anti-tumor immune responses. Researchers have shown that several chemotherapeutic drugs increase tumor-specific CD8+ T-cell infiltration and tsMHC-I expression *in vitro* studies involving different tumor cell types. Even though it does not cause immunogenic cell death *in vitro*, the popular chemotherapeutic drug docetaxel may enhance the expression of antigen presentation pathway components like TAP and Tsn (205). When tumor cells were treated with docetaxel, neither ATP nor high-mobility group box 1 (HMGB1) production nor cell death was seen. Nevertheless, after chemotherapy, CRT exposure was seen in every cell line analyzed. Following docetaxel therapy, killing by CEA, MUC-1, or PSA-specific CD8+ CTLs was markedly increased. Functional knockdown of CRT, PERK, or CRT-blocking peptide revealed that this killing was primarily caused by CRT membrane translocation and was linked to increases in antigen-processing machinery components. Continuous treatment with docetaxel resulted in the selection of a docetaxel-resistant cell line (MDR-1^+^, CD133^+^). These cells were not immune to the chemomodulatory effects that increased CTL killing, even if they were resistant to the direct cytostatic impacts of docetaxel. Here, scientists provide an operational description of “immunogenic modulation,” which is the process by which tumor cells exposed to nonlethal or sublethal chemotherapeutic dosages change their phenotypic, making the tumor more vulnerable to CTL destruction. Researchers provided a manner by which chemotherapy and immunotherapy may be utilized in conjunction, and they are different from and complementary to immunogenic cell death (233).

Allogeneic CAR-T cells may address autologous T-cells’ low quantity and poor quality. When HLA divergence occurs, the host immune system rejects allogeneic T-cells. Generating negative HLA T-cells through the CRISPR/Cas9 could address this issue. The activation of NK cells is a primary factor contributing to the mortality of CAR-T cells, potentially initiated by negative MHC-I interactions. Consequently, additional analysis is necessary to identify methods to inhibit their ability to eliminate CAR-T cells (234).

The adaptive immune system must be strongly activated for peptide-based cancer vaccines to exert their effector role. Although they are very safe and specific, they have not yet been demonstrated to be a successful cancer therapy in clinical settings. Tumor heterogeneity, self-tolerance, and immune suppression are a few causes of this. The general architecture of peptide-based cancer vaccines has been given significant attention. These vaccines have progressed from basic peptide derivatives of a cancer antigen to sophisticated medications that use conjugates, overlapping regions, and delivery mechanisms to target and activate various APC components and enhance antigen cross-presentation. Peptide-based cancer vaccinations are often used in conjunction with current cancer therapies and are getting more and more tailored to a person’s repertoire of tumor antigens (235–239). Ultimately, this approach helps counteract the drawbacks of a more universal vaccination. It offers a thorough course of therapy that considers the variety of cancer cells and their capacity to evade immune interrogation. Furthermore, as of May 2021, there were about 80 phase I or II clinical studies using a peptide-based vaccination method for cancer; 20 were ongoing at the time of writing, and another 20 had been completed since the beginning of 2019. summarizes the ongoing Phase I and II peptide-based cancer vaccination studies recruiting or actively taking participants. Peptide-based cancer vaccines are high on the list; they protect against blood, brain, lung, and BC, to mention a few. Researchers were demonstrating the variety of targets being tested for peptide-based cancer vaccines. The absence of phase II studies, however, is visible and highlights the present problems with effectiveness that peptide-based cancer vaccines encounter. Nonetheless, there is a positive trend in the pool of ongoing studies towards a more individualized approach to patient neoepitope selection, with a stronger emphasis on using peptide-based cancer vaccines in conjunction with other cancer therapy approaches. Bezu L. et al. have meticulously compiled and examined studies for peptide-based cancer vaccines until 2018 to provide a more thorough study (240–242).

The above information suggests that peptide vaccinations may stimulate robust CD8+ Tcell responses, which can sometimes help patients clinically. Vaccines based on peptides provide many benefits as a cancer immunotherapy approach. The flexible architecture of these vaccines enables the inclusion of multiple peptide epitopes within a single dose. This facilitates the incorporation of numerous MHC-I epitopes that provoke a T-cell response. It is crucial to include peptides that bind to single alleles (like HLA-A2 or HLA-A24) and those that can bind to multiple alleles (like HLA-A2 and HLA-A24) in the same formulation since people have different MHC alleles. This suggests that people with a wide range of MHC allele variations may benefit from a vaccination made from peptides that have been naturally processed. Furthermore, by eliciting a more diffuse and oligoclonal immune response, a vaccination with many epitopes may be able to prevent tumors from developing resistance to antigen downregulation. Although many epitopes from the same antigen may evade HLA restriction (e.g., MAGE-n, survivin, and CEA), the epitopes used in this kind of vaccination must come from different parent proteins. This method will prevent tumor cells from reducing the expression of a specific protein, which will increase the number of clonal T-cells and prevent them from responding to the vaccination. Peptide-based vaccines have the potential to include both B cell and MHC-II-restricted epitopes, which may activate CD4+ T-cells and T helper and antibody-mediated responses, respectively. A more dependable and effective way of eliminating cancers may be possible with a combined complete adaptive immune response. Antigen processing differs substantially among cells even though there are similar reasons why a protein-based vaccine might be attractive (243) (**Table 3**).

**Table 3:** A brief overview of some prominent cancer neoantigens together with the HLA alleles, sequences, and possible uses for which they are linked.

Cancer neoantigen	Sequences	Associated HLA	Potential applications	Landscape	Ref
KRAS G12D	GADGVGKSA	HLA-A*11:01	Personalized cancer vaccines and TCR-T cell treatments can target this patient. The affinity-improved TCR may also be utilized to target cancer cells that express KRAS G12D by fusing it to a humanized anti-CD3 scFv.	Cancers of the pancreas, colon, and lungs often have mutations in this gene. There is great promise for the use of immunotherapy to treat KRAS mutations.	(244, 245)
KRAS G12D	GADGVGKSA	HLA-C*08:02	In a patient with metastatic colorectal cancer, researchers found tumor-infiltrating lymphocytes that targeted KRAS G12D with TCRs that were confined to HLA-C*08:02. This patient’s metastatic pancreatic cancer objectively regressed after receiving TCR gene therapy that targeted the KRASG12D driving mutation.	Pancreatic cancer and other malignancies expressing KRAS G12D should be the subjects of prospective clinical studies to ascertain the therapy’s therapeutic potential.	(246)
KRAS G12V	VVGAVGVGK	HLA-A*11:01	Two TCRs that are unique to the KRAS G12V-9 peptide and modified TCR-T cells that showed specific responses to different types of tumor cells with the KRAS-G12V mutation have been developed. When combined with anti-PD-1 antibodies, the 1-2C TCR-T cells demonstrated enhanced anti-tumor potency and successfully suppressed tumors *in vivo*.	To better target the KRAS G12V mutant in future treatment designs, it would be helpful to understand the structural basis for its unique presentation and particular identification. Therefore, researchers presented a viable treatment option for malignancies with the common KRAS G12V mutation.	(247)
TP53 R175H	HMTEVVRHC	HLA-A*02:01	Looks good for the future of neoantigen vaccines, particularly for TP53-mutated malignancies. A tumor-specific CD3+ T cell receptor that targets the HLA-A*02:01-restricted p53R175H neoantigen; the molecular basis for this antibody’s specificity; and its ability to transform into a bispecific antibody that can lyse cancer cells when the neoantigen is present.	This treatment seems to be very selective to the mutation-carrying cancer cells. Because TP53 mutations are common in a variety of malignancies, they serve as an immunogenic hotspot for research on neoantigens. Additionally, compared to cell-based treatments, protein-based therapies have the benefit of being “off-the-shelf,” being much simpler to create, and being far less costly.	(248)
TP53 R248Q	KGETFRYLP	HLA-A*24:02	In high-grade serous ovarian cancer (HGSOC), the overexpression of the gain-of-function p53 mutation (p53R248Q) may impact EGFR-related signaling and the resulting medication inhibition effect. Under EGFR/MDM2-targeted inhibition, the R248Q mutation of p53 in HGSOC resulted in notable alterations in signaling protein activity and trafficking.	If the R248Q mutation of p53 is found in HGSOC patients, this model may change the therapeutic strategy for cancer therapy, which recommends using either gefitinib or JNJ alone; if not, the combination of gefitinib and JNJ should be advised.	(249)
EGFRvIII	VSRGEEKKK	HLA-A*02:01	A new target for humoral and cell-mediated immunotherapy in a range of malignancies is the tumor-specific EGFRvIII mutation. extensively researched in modified T cell treatments and glioblastoma vaccinations. It is a primary target for glioblastoma treatment since it is only expressed in glioblastoma and not in healthy tissues.	By looking for additional immunogenic peptides, adding more potent adjuvants, or combining them with other immunotherapies like ICIs, the effectiveness of EGFR-targeting peptide vaccines may be further increased in the future. The discovery of common NeoAgs also makes room for further immunotherapeutic strategies, such as treatments based on TCRs.	(250, 251)
BRAF V600E	KIGDFGLATEK	HLA-A*03:01 and HLA-A*11:01	BRAFV600E is one example of a non-synonymous mutation that may produce neoantigens that effectively stimulate antitumor CD8+ T cell responses. HLA-I molecules do not contain HLA-I binding or proteasome-generated neopeptides formed from the BRAFV600E protein.	Researchers reported raises concerns about the effectiveness of immune checkpoint inhibitor treatment in Langerhans cell histiocytosis (LCH) as the BRAFV600E mutation is quite common in individuals with chemotherapy-refractory LCH who may be eligible for immunotherapy.	(252)
GNAQ Q209	YVIDYYRKPI	HLA-A*02:01	T-cell reactions may be triggered by recurrent mutations in the *GNAQ* and *GNA11* genes. Uveal melanoma (UM) may be a desirable target for immunotherapeutic treatments due to the anticipated low immune selective pressure in this condition. Furthermore, researchers speculated that distinct mutations (*Q209P* or *Q209L*) may exhibit varying antigenicity and immune system responses.	Researchers suggested a high potential immunogenicity of the GNAQ/11 Q209L variant that could allow the generation of novel therapeutic tools to treat UM like neoantigen vaccinations. Q209L mutation is presented in the MHC context and detected by T cells. Although preliminary, Researchers paved the way for future therapeutic options in uveal melanoma patients.	(253)
MAGE-A1	EADPTGHSY	HLA-A*02:01	Investigated for melanoma and other malignancies using vaccine-based methods.	It is a tumor-associated antigen as it is expressed in a variety of cancer types while being hardly noticeable in healthy tissues.	(254)
IDH1 R132H	HMGYVGAE	HLA-A*03:01	target in glioma immunotherapy, especially in formulations of customized vaccines. The lack of humoral and cellular reactions in the blood and tumors of patients with lower-grade gliomas (LGGs) suggested that IDH1 R132H is not immunogenic enough and discourages its potential for future therapeutic use, at least in most LGG patients.	Glioma vaccination studies are being driven by a neoantigen that is present in gliomas and cholangiocarcinomas.	(255–257)

## 10 Conclusion

The production of pMHC-I complexes, which are necessary for identifying cancer cells, is coordinated by the PLC and presented on the cell surface. Cancer cells employ various strategies to evade detection by the immune system, including suppressing MHC-I molecule synthesis and other proteins that play a role in the processing and presentation of antigens. They also employ various strategies to inhibit the expression of TAP, Tsn, CRT, ERp57, MHC HC, or β_2_m, resulting in the diminished or absent expression of MHC-I on the cell surface. The accuracy and side effects of cancer treatment approaches are reduced, and medicines are advanced due to understanding the impact of PLC components on different forms of cancer. Furthermore, vaccinations based on peptides provide several benefits as a cancer immunotherapy strategy. These vaccines have a versatile architecture that allows them to include many peptide epitopes in a single dosage. This makes adding many MHC-I epitopes that trigger a T-cell response possible. The absence of a comprehensive and coordinated mobilization of the MHC system is a fundamental drawback of existing protein-based standalone vaccination methods, which often results in an inability to produce potent anti-tumor therapeutic effects. The vaccine’s clinical restriction may be addressed by concentrating research on the role of the PLC component in various cancer types. As a result, more thorough research on all cancer types—including blood, lung, prostate, and BC—should be conducted.
